# Cost and availability of selected medicines after implementation of increased import verification fees

**DOI:** 10.1186/s12913-023-10433-7

**Published:** 2024-01-04

**Authors:** Helen Byomire Ndagije, Diana Nakitto Kesi, Kalidi Rajab, Solomon Onen, Allan Serwanga, Leonard Manirakiza, Sheila Ampaire, Joseph Mutasaaga, Denis Mwesigwa, David Nahamya

**Affiliations:** 1 National Drug Authority, Plot 46-48 Lumumba Avenue, P.O.Box 23096, Kampala, Uganda; 2 Uganda National Bureau of Standards, Plot 2-12 By pass Link Bweyogerere Industrial and Business Park, Kampala, Uganda; 3 Department of Pharmacy, School of Health Sciences, College of Health Sciences, Makerere University, University Rd, 10218 Kampala, Uganda

**Keywords:** Import verification fees, Availability of medicines, Cost of medicines, Local products, Imported products

## Abstract

**Background:**

Uganda imports approximately 90% of its medicines, with about 60% being distributed by the private sector. To discourage importation and promote local production of 37 selected locally manufactured medicines, the Ugandan government through the Ministry of Health in 2017 increased the import verification fees from 2 to 12%. The increase in verification fees ultimately affects cost and availability of these medicines. This study aimed to assess the cost and availability of the selected essential medicines after the 12% increase in verification fees in Uganda.

**Methods:**

A cross sectional study among 328 wholesale and retail pharmacies and seven key informant interviews was conducted using a pretested data collection checklist and in-depth interview guide from February to September 2021 in Uganda. Data on the availability and prices of the medicines before (2017) and after (2020) the increase in verification fees was collected. Paired sample T-Test was used to test if there is a significant difference in prices before and after the 12% increase in verification fees.

**Results:**

Mean availability of imported medicines was higher (54.8%, CI: 49.3–60.4) than the locally produced medicines (37.1%, CI: 31.9–42.7) except for locally manufactured parenteral preparations (54.6.%, CI: 49.1–60.1). Availability of locally produced medicines was mainly low (45%) while the imported medicines were fairly high (74%). Most commonly available locally manufactured medicines were Surgical spirit (89.9%), ORS (86%), Dextrose 5% solution (74.4%), Paracetamol 500 mg Tablets (73.8%) and Sodium Chloride 0.9% solution (72.9%). Most commonly available imported medicines were; Omeprazole 20 mg (94.2%), Amoxicillin Trihydrate 125 mg/5 ml (92.4%), Ciprofloxacin 500 mg (91.4%), Paracetamol Suspension 120 mg/5 ml (91.5%) and Metronidazole 200 mg Tablets (88.1%). Increase in lowest-priced local and imported medicines was significant for 10 (23.8%) and 7 (15.9%) of the medicines respectively. The median prices of imported medicines were generally higher than locally produced medicines. The median unit prices of 12 (28.6%) locally produced medicines and 20 (47.6%) imported medicines were higher than the international median unit prices.

**Conclusions:**

The overall availability of imported medicines was still higher than the local medicines. The median prices of local and imported medicines generally increased or remained the same after the introduction of import verification fees. There is a need for price controls and transparency in the private sector.

**Supplementary Information:**

The online version contains supplementary material available at 10.1186/s12913-023-10433-7.

## Background

The demand for safe, effective and affordable medicines has increased over the years with the increasing number of communicable and non-communicable diseases. Local production of essential medicines is promoted as one of the ways to ensure the supply of quality assured safe, efficacious and affordable medicines. Local pharmaceutical production contributes to prevention of medicine stock outs, promotes local value addition, reduces medicine costs, generates income by creating jobs, promotes self-reliance and is a step towards sustainability of treatment programs and maintaining access to medicines beyond the era of drug donations [1]. It is estimated that Africa imports around 79% of all pharmaceuticals [2]. In Uganda, majority share (90%) of essential medicines and health supplies are imported and about 60% are distributed through the private sector [3]. The Pharmaceutical market in Uganda is mainly dominated by imports [4].

In an attempt to reverse the trend of overreliance on importation, the Ugandan government introduced the Buy Uganda Build Uganda (‘BUBU”) [5] policy and increased import verification fees for 37 selected locally manufactured essential medicines from 2% to 12 [6]. This well-intentioned policy to promote local pharmaceutical production may however have an unintended negative impact on access to medicines. Tariffs are vital determinants of prices and they can considerably increase the prices of imported goods with subsequent reduction in affordability and availability of the medicines to the final consumer [7]. The final price of medicines is affected by its importation costs, its fee on board costs, its wholesale markup and its retail markup factors [8]. The latter three determinants are uniform for both locally manufactured and imported medicines while the former first determinant is incurred for only imported medicines. Therefore, in addition to promoting local production, import verification fees will also affect the final price of medicines hence affordability and availability.

The National medicine policy in Uganda like in many other countries aims to contribute to the attainment of a good standard of health for the population through ensuring the availability, accessibility and affordability at all times of essential drugs of appropriate quality, safety and efficacy, and by promoting their rational use [9]. As governments introduce policies to discourage importation and promote local production, the policies should as well increase access to essential medicines. The import verification fees on 37 selected essential medicines was increased from 2 to 12% effective August 1st 2017 [10]. To date there is no data on the impact of the increment on availability and cost of both imported and locally produced medicines. This study assessed the impact of the verification fees increase on the cost and availability of the 37 selected essential medicines. This is critical as the country and other developing countries seek to adopt similar strategies to promote local production and at the same time ensure affordability and accessibility of medicines.

## Methods

### Study aim, design, setting and population

This study assessed the impact of the 12% verification fees on cost and availability of selected essential medicines in Uganda. This was a cross sectional study and both qualitative and quantitative data were collected. Quantitative data were collected using a checklist. An in-depth interview guide was used to collect qualitative data from Key informants. The study was carried out in the seven (7) National Drug Authority (NDA) regions of Uganda i.e. Central, Eastern, South Eastern, Northern, West Nile, Western and South Western. The study included wholesale and retail pharmacies in each of these regions. By 2019, Uganda had a total of 1,735 registered pharmaceutical outlets according NDA management information System. These included 539 wholesale pharmacies and 1196 retail pharmacies [11]. The study included licensed wholesale and retail pharmacies that existed for at least five years and the managers of the manufacturing facilities and other key informants from relevant Ministries, Departments, Agencies and pharmaceutical importers. This study was part of a bigger study that also assessed the impact of the verification fees on local production capacity of pharmaceuticals.

### Sample size and sampling procedure

Using the Slovin’s formula for sample size calculations, n = N/ (1 + Ne²), the calculated minimum sample size for the pharmacies was 326. The total number of wholesale and retail pharmacies N = 1735, e = maximum allowable error = 5%. Sample size n = 1735/ (1 + 4.337) n = **326** (number of wholesale and retail pharmacies). The number of wholesale and retail pharmacies and their distribution in the different regions was proportionately determined as shown in Tables 1 and 2. In each region, a city and two other districts were selected. The two other districts were selected based on the existing number of pharmacies in each district in the region. Furthermore, the pharmacies selected were those that had been in existence for more than five (5) years. These were selected by simple random sampling. For regions with more than one city, simple random sampling was used to select the city. For the selected districts and cities, the number of the pharmacies was also proportionately determined. For each district, the pharmacies were randomly selected. Excel was used to generate the random sample of pharmacies.

**Table 1:** Sample Distribution of whole sale and retail pharmacies in the NDA regions

Region	Total number Whole sale	Total number Retail	Sample Wholesale pharmacies	Sample retail pharmacies
Central-Nakawa	276	841	52	159
Southwestern-Mbarara	58	90	11	17
West Nile-Arua	12	19	2	4
Western-Hoima	53	63	10	12
Northern-Lira	33	36	6	7
Eastern-Tororo	35	50	7	9
South eastern-Jinja	72	97	13	18
	**539**	**1196**	**101**	**227**

**Source;** NDA management information systems 2019

The final sample size used in this study was n = 328.

The key informants were purposively selected to participate in the study. These included 7 Key informants from 2 pharmaceutical industries manufacturing any of the selected 37 essential medicines, 1 from Uganda Pharmaceutical manufacturers association,1 from Uganda Pharmacy owners Association, 1 from an importing company of the selected medicines and 2 from the central medical stores.

### Data collection methods and tools

At the retail and wholesale pharmacies, quantitative data were collected using a pre-tested data checklist. Information about the cost and availability of the selected medicines were obtained. The In-depth semi-structured interview guide that was used for the key informant interview was developed from available literature (S11). The key informant interviews lasted 45–60 min and were conducted by the principal investigator with the help of a research assistant. Through the interviews, the views and perceptions of the respondents was sought regarding the impact of the increase in verification fees on cost and availability of the medicines and any challenges and recommendations regarding the import verification fees increment.

### Data management and quality control

Data was cleaned to ensure that all relevant and correct data were collected. The principal investigator (PI) and study team oversaw accuracy and completeness of all data entered on the checklist before submission for data entry. A web based Open Data Kit (ODK) tool was used for data entry and a number of range checks, logical skips, missing data checks were incorporated at data entry level. The data sets entered were cross referenced and errors, inconsistencies were resolved by checking against the source documents after which one data set was produced. No names were used; identification codes were allocated to each facility checklist. Pre-testing of data collection tools was done in five pharmacy outlets. The in-depth interviews were audio recorded and transcribed later to ensure all information given by respondents were not lost. In addition to audio records, notes were also taken during the interviews. Data collection was done by trained research assistants supervised directly by a member of the lead research team. Computers used for data entry were password protected.

### Data analysis

The quantitative dataset was transferred from the ODK tool to Microsoft Excel 2017 for further cleaning and validation. Data analysis was done using SPSS V25.0. The median prices before and after the introduction of the 12% increase were computed. Paired sample T-Test was used to test if there is a significant difference in these prices before and after the 12% increase. International reference unit prices were obtained from Management Sciences for Health (MSH) International Medical Products Price Guide 2015 [12]. For conversions, 1 US$ = 3670 Ugandan Shillings (Uganda Revenue Authority (URA) median exchange rate from 2017 to 2020). Price adjustment for inflation/deflation was taken at 5.63 in 2017 (Uganda Bureau of Statistics (UBOS) Consumer Price Index (CPI) Publication, 2017) and 3.79 in 2020 (UBOS CPI Publication, 2021). The international comparisons of median unit prices were computed using median price ratios (MPR) as follows;

$$Medicine\, Price\, Ratio \left(MPR\right)=\frac{Median\, local\, unit\, price}{International\, reference\, unit\, price}$$

Using the Consumer Price Indices (CPI) for 2017 and 2020, the median unit prices were adjusted for inflation/deflation using the following formula.

$$Percentage\, Inflation\, rate= \frac{CPI \left(2020\right)-CPI\left(2017\right)}{CPI\, \left(2017\right)}X 100$$

$$Percentage\, Inflation\, rate= \frac{3.79-5.63}{5.63}X 100 = 33\%$$

Therefore, a deflation rate of 33% was used to adjust the median unit prices.

Availability of medicines were captured as the average percentage of the medicines that were available at the time of data collection. These were aggregated to get the average availability of all the drugs by region, Vital, Essential and Necessary (VEN) classification and by formulation and these were compared across the pharmacy type [Retail, wholesale and dual (both retail and wholesale)]. Analysis of Variance (ANOVA) was used to test the difference in the average availability and whether there was a difference in availability by region, VEN classification and by formulation. Availability of medicines was also categorized based on WHO categorization into Very low (< 30%), Low (30–49%), fairly high (50–80%) and high (> 80%).

The qualitative data were thematically analyzed following the steps proposed by Braun and Clarke which included transcription, coding of the data, and generation, reviewing and naming of themes [13]. The voice records were transcribed verbatim as text and the transcripts were then compared with the notes taken during the interviews and any missing information due to unclear audio recordings were added. The next step was to read the transcript so as to get familiar with all responses from the interviews. Meanings were then generated from transcribed data, during a second perusal of the transcribed data, following which emergent sub themes were developed. This was then followed by categorization and alignment of each of the quotes with their respective sub themes, and finally, with their predetermined themes. Precedence of a given response was established based on the recurrence of responses. Emerging quotes from the interviews were highlighted and marked for referencing and reported verbatim.

## Study results

### Pharmacy and key informant characteristics

This study involved 328 pharmacy outlets and seven key informants. The key informants included 2 members of the Uganda pharmaceutical manufacturers association, 2 people from the central medical stores, 2 members of the Uganda pharmacy owners’ association and 1 person from a pharmaceutical importing company.

The characteristics of surveyed pharmacies are presented in Table 2. Retail pharmacies accounted for a large proportion (n = 223, 68.0%) of the pharmacies and majority of the surveyed pharmacies had been in existence for 5 to 10 years (n = 256, 78.0%).

**Table 2:** Showing pharmacy characteristics

Characteristics	Frequency (n = 328)	Percentage (%)
**Pharmacy location (NDA region)**		
Central-Nakawa	212	64.6
Southwestern-Mbarara	28	8.5
West Nile-Arua	6	1.8
Western-Hoima	24	7.3
Northern-Lira	13	4.0
Eastern-Tororo	15	4.6
South eastern-Jinja	30	9.1
**Pharmacy type**		
Wholesale	99	30.2
Retail	223	68.0
Dual	6	1.8
**Number of years in existence**		
5–10	256	78.0
> 10	72	22.0

### Availability of the selected essential medicines after the increment of import verification fees

#### Mean availability of local manufactured and imported medicines

The overall mean availability of locally manufactured medicines (37.1%, CI: 31.9–42.7) was lower than that of imported medicines (54.8%, CI: 49.3–60.4). The Eastern-Tororo region had the highest (45.2%, CI: 39.6–50.7) overall availability of locally manufactured medicines while the West Nile- Arua had the lowest (29.9%, CI: 25.0-35.2) overall availability. The overall availability of imported medicines was highest (60.4%, CI: 54.8–65.7) in South-Eastern Jinja region and lowest (42.6%, CI: 37.3–48.2) in Western-Hoima region. When compared using ANOVA, there was a significant difference in the availability of both locally manufactured and imported medicines across the regions (p < 0.0001). By VEN classification, most of the available locally manufactured medicines (55.7%, CI: 50.1–61.3) fall under the Necessary category while the availability of imported Necessary items was low (43.6%, CI: 41.4–48.0). There was a significant difference in the availability of medicines across the three classification categories (p < 0.0001). By formulation, locally manufactured parenteral preparations (54.6%, CI: 49.1–60.1) were available in most of the pharmacies. For imported formulations, tablets/capsule (63.9%, CI: 60.7–70.3) were mostly available while parenteral preparations (23.6%, CI: 22.4–26.0) were least available. Details in (Table 3).

**Table 3:** Mean availability of local and imported medicines

Characteristics		% of Local brands (95% CI)	*P* value	% of Imported brands (95% CI)	*P* value
Region	Central-Nakawa	31.9 (27.0-37.4)	< 0.0001	57.0 (51.5–62.4)	< 0.0001
	South Western-Mbarara	35.5 (30.2–40.8)		50.8 (45.4–56.4)	
	West Nile-Arua	29.9 (25.0-35.2)		56.1 (50.5–61.5)	
	Western-Hoima	43.2 (37.9–48.8)		42.6 (37.3–48.2)	
	Northern-Lira	36.5 (31.4–42.1)		57.7 (52.1–63.0)	
	Eastern-Tororo	45.2 (39.6–50.7)		59.2 (53.6–64.5)	
	South eastern-Jinja	37.8 (32.5–43.3)		60.4 (54.8–65.7)	
	**Overall availability**	**37.1 (31.9–42.7)**		**54.8 (49.3–60.4)**	< 0.0001
VEN classification	Vital	33.7 (30.3–37.1)	< 0.0001	57.4 (54.5–63.1)	
	Essential	27.9 (25.1–30.7)		57.4 (54.5–63.1)	
	Necessary	55.7 (50.1–61.3)		43.6 (41.4–48.0)	
					0.0012
Formulation	Tablets/capsules	35.8 (32.2–39.4)	0.0011	63.9 (60.7–70.3)	
	Oral suspensions/syrups	21.5 (19.4–23.7)		61.7 (58.6–67.9)	
	Parenteral preparations	54.6 (49.1–60.1)		23.6 (22.4–26.0)	

#### Availability of medicines in different pharmacy outlets by WHO categorization

Overall, many of the pharmacies had low (45%) and very low (41%) availability for locally manufactured medicines and fairly high (74%) availability for the imported medicines. None of the pharmacies in the different regions recorded high availability of locally manufactured medicines. In central region, medicine availability of locally manufactured medicines was very low in most of the pharmacies (49.3%) while the imported medicines were fairly high (76.9%). In Western region, availability of locally manufactured medicines was fairly high in surveyed pharmacies (45.8%) and the imported medicines were low (54.2%). Details in Table 4.

**Table 4:** Availability of medicines in pharmacy outlets by WHO categorization

Source	Characteristics		Pharmacies n (%)			
			Very low	Low	Fairly high	High
Local	Region	Central-Nakawa	104 (49.3%)	91 (43.1%)	16 (7.6%)	0 (0%)
		Southwestern-Mbarara	10 (35.7%)	15 (53.6%)	3 (10.7%0	0 (0%)
		West Nile-Arua	2 (33.3%)	4 (66.7%)	0 (0.0%)	0 (0%)
		Western-Hoima	5 (20.8%)	8 (33.3%)	11 (45.8%)	0 (0%)
		Northern-Lira	5 (38.5%)	5 (38.5%)	3 (23.1%)	0 (0%)
		Eastern-Tororo	0 (0.0%)	10 (66.7%)	5 (33.3%)	0 (0%)
		South eastern-Jinja	8 (26.7%)	15 (50.0%)	7 (23.3%)	0 (0%)
		**Overall (average)**	134 (41.0%)	148 (45.3%)	45 (13.8%)	0 (0.0%)
Imported	Region	Central-Nakawa	9 (4.2%)	33 (15.6%)	163 (76.9%)	7 (3.3%)
		Southwestern-Mbarara	0 (0.0%)	13 (46.4%)	15 (53.6%)	0 (0.0%)
		West Nile-Arua	0 (0.0%)	1 (16.7%)	5 (83.3%)	0 (0.0%)
		Western-Hoima	4 (16.7%)	13 (54.2%)	7 (29.2%)	0 (0.0%)
		Northern-Lira	1 (7.7%)	1 (7.7%)	11 (84.6%)	0 (0.0%)
		Eastern-Tororo	0 (0.0%)	1 (6.7%)	14 (93.3%)	0 (0.0%)
		South eastern-Jinja	0 (0.0%)	4 (13.3%)	26 (86.7%)	0 (0.0%)
		Overall (average)	14 (4.3%)	66 (20.1%)	241 (73.5%)	7 (2.1%)

#### Proportion and average number of imported and locally manufactured medicines available

Generally, 65% of the brands of the 37 selected medicines available in the surveyed pharmacies were imported. The average number of locally manufactured generics in the pharmacies were highest (21) in Eastern region while lowest (14) in West Nile region. There was a significant difference in average number of locally manufactured generics in pharmacies across the regions (F = 10.384, P < 0.0001). The average number of imported generics and originator brands in the pharmacies were highest (37) in South Eastern region and lowest in Western region (23). There was a significant difference in average number of imported generics and originator brands in pharmacies across the regions (F = 7.015, P < 0.0001). By pharmacy type, the average numbers of locally manufactured generics was highest (21) in dual pharmacies while retail pharmacies had the highest (36) average number of imported generics and originator brands. There was a significant difference in the number of locally manufactured generics (F = 6.986, P = 0.0132) and number of imported generics and originator brands across the pharmacy types (F = 23.467, *P* < 0.0001) (Table 5).

**Table 5:** Average number of locally manufactured generics and imported generics and originator brands

Characteristics	Local generics (n)	F-Statistic	*P*-Value	Imported generics and originator brands (n)	F-Statistic	*P* value
Region					7.015	< 0.0001
Central-Nakawa	15	10.384	< 0.0001	35		
Southwestern-Mbarara	16			28		
West Nile-Arua	14			28		
Western-Hoima	19			23		
Northern-Lira	17			33		
Eastern-Tororo	21			35		
South eastern-Jinja	17			37		
Pharmacy type					23.467	< 0.0001
Wholesale	18	6.986	0.0132	29		
Retail	15			36		
Dual	21			25		

#### Individual availability of medicines

The top five (5) most commonly available locally manufactured medicines included; Surgical spirit (89.9%), ORS (86%), Dextrose 5% solution (74.4%), Paracetamol 500 mg Tablets (73.8%), Sodium Chloride 0.9% solution (72.9%). The least five (5) available locally produced medicines were; Artemether / Lumefantrine 15/90 Dry Suspension (0.9%), Omeprazole 20 mg (0.9%), Albendazole Suspension 100 mg/5 ml (1.2%), Cetirizine Hydrochloride 10 mg Tablets (3%), Cloxacillin 125 mg/5 ml (4%). Very few 2 (4.7%) of the medicines met WHO target of 80% availability. Table 6 and S1.

The five (5) most commonly available imported medicines were; omeprazole 20 mg (94.2%), Amoxicillin Trihydrate Equivalent To Amoxicillin 125 mg/5 ml (92.4%), Ciprofloxacin 500 mg (91.4%), Paracetamol Suspension 120 mg/5 ml (91.5%) and Metronidazole 200 mg Tablets (88.1%) while the least five (5) available imported medicines were ORS + Zinc Sulphate Monohydrate 20 mg Tablets (0.3%), surgical spirit (1.8%), Zinc Solution Supplement 10 mg/5 ml (4.3%), Hartmann’s Ringers Lactate solution 49(14.9%) and Dextrose 50% solution 57 (17.4%). Only 11 (25.6%) of the medicines met the WHO target of 80% availability. Table 7 and S2.

**Table 6:** Selected locally manufactured medicine availability by region and overall availability in all regions

Brand Names	West Nile-Arua	Western-Hoima	South eastern-Jinja	Northern-Lira	Southwestern-Mbarara	Central-Nakawa	Eastern-Tororo	Overall
Surgical Spirit	5(83.3%)	20(83.3%)	26(86.7%)	12(92.3%)	25(89.3%)	192(90.6%)	15(100.0%)	295(89.9%)
ORS	5(83.3%)	20(83.3%)	26(86.7%)	10(76.9%)	22(78.6%)	187(88.2%)	12(80.0%)	282(86.0%)
Dextrose 5% solution	3(50.0%)	20(83.3%)	27(90.0%)	8(61.5%)	23(82.1%)	150(70.8%)	13(86.7%)	244(74.4%)
Paracetamol 500 mg Tablets	4(66.7%)	22(91.7%)	24(80.0%)	9(69.2%)	27(96.4%)	143(67.5%)	13(86.7%)	242(73.8%)
Sodium Chloride 0.9% solution	4(66.7%)	19(79.2%)	24(80.0%)	9(69.2%)	24(85.7%)	146(68.9%)	13(86.7%)	239(72.9%)
Hartmann’s Ringers Lactate solution	4(66.7%)	20(83.3%)	25(83.3%)	7(53.8%)	22(78.6%)	128(60.4%)	13(86.7%)	219(66.8%)
Chloramphenicol Palmitate 125/5 ml	2(33.3%)	8(33.3%)	2(6.7%)	5(38.5%)	7(25.0%)	37(17.5%)	7(46.7%)	68(20.7%)
Mannitol 20%	0(0.0%)	5(20.8%)	13(43.3%)	2(15.4%)	14(50.0%)	29(13.7%)	3(20.0%)	66(20.1%)
Ibuprofen Suspension 100 mg/5 ml	2(33.3%)	8(33.3%)	8(26.7%)	5(38.5%)	6(21.4%)	30(14.2%)	5(33.3%)	64(19.5%)
Metronidazole Suspension 100 mg/5 ml	1(16.7%)	9(37.5%)	7(23.3%)	2(15.4%)	2(7.1%)	40(18.9%)	2(13.3%)	63(19.2%)
Sulfamethoxazole 200 mg / Trimethoprim 400 mg / 5 ml	1(16.7%)	10(41.7%)	6(20.0%)	3(23.1%)	6(21.4%)	27(12.7%)	8(53.3%)	61(18.6%)
Ampicillin 125 mg + Cloxacillin 125 mg/5 ml	0(0.0%)	12(50.0%)	4(13.3%)	1(7.7%)	5(17.9%)	31(14.6%)	5(33.3%)	58(17.7%)
Ciprofloxacin 500 mg	3(50.0%)	5(20.8%)	2(6.7%)	1(7.7%)	2(7.1%)	30(14.2%)	4(26.7%)	47(14.3%)
Amoxicillin Trihydrate Equivalent To Amoxicillin 125 mg/5 ml	0(0.0%)	8(33.3%)	4(13.3%)	2(15.4%)	4(14.3%)	25(11.8%)	3(20.0%)	46(14.0%)
ORS + Zinc Sulphate Monohydrate 20 mg Tablets	0(0.0%)	1(4.2%)	5(16.7%)	2(15.4%)	1(3.6%)	23(10.8%)	4(26.7%)	36(11.0%)
Paracetamol Suspension 120 mg/5 ml	0(0.0%)	8(33.3%)	5(16.7%)	0(0.0%)	1(3.6%)	19(9.0%)	0(0.0%)	33(10.1%)
Diclofenac Sodium 100 mg	0(0.0%)	2(8.3%)	1(3.3%)	0(0.0%)	6(21.4%)	17(8.0%)	5(33.3%)	31(9.5%)
Albendazole Suspension 100 mg/5 ml	0(0.0%)	0(0.0%)	0(0.0%)	0(0.0%)	1(3.6%)	3(1.4%)	0(0.0%)	4(1.2%)
Artemether / Lumefantrine 15/90 Dry Suspension	0(0.0%)	0(0.0%)	2(6.7%)	0(0.0%)	0(0.0%)	1(0.5%)	0(0.0%)	3(0.9%)
Omeprazole 20 mg	0(0.0%)	1(4.2%)	1(3.3%)	0(0.0%)	0(0.0%)	1(0.5%)	0(0.0%)	3(0.9%)

**Table 7:** Selected imported medicine availability by region and overall availability in all regions

Brands	West Nile-Arua	Western-Hoima	South eastern-Jinja	Northern-Lira	Southwestern-Mbarara	Central-Nakawa	Eastern-Tororo	Overall
Omeprazole 20 mg	4(66.7%)	22(91.7%)	28(93.3%)	11(84.6%)	28(100.0%	201(94.8%)	15(100.0%)	309(94.2%)
Amoxicillin Trihydrate Equivalent To Amoxicillin 125 mg/5 ml	6(100.0%)	20(83.3%)	29(96.7%)	12(92.3%)	26(92.9%)	197(92.9%)	13(86.7%)	303(92.4%)
Ciprofloxacin 500 mg	4(66.7%)	22(91.7%)	30(100.0%)	13(100.0%)	26(92.9%)	192(90.6%)	14(93.3%)	301(91.8%)
Paracetamol Suspension 120 mg/5 ml	6(100.0%)	21(87.5%)	29(96.7%)	13(100.0%)	26(92.9%)	190(89.6%)	15(100.0%)	300(91.5%)
Metronidazole 200 mg Tablets /	4(66.7%)	20(83.3%)	26(86.7%)	9(69.2%)	27(96.4%)	189(89.2%)	14(93.3%)	289(88.1%)
Amoxicillin Trihydrate Equivalent To Amoxicillin 250 mg	5(83.3%)	17(70.8%)	29(96.7%)	10(76.9%)	24(85.7%)	185(87.3%)	14(93.3%)	284)86.6%
Albendazole 400 mg Tablet	3(50.0%)	15(62.5%)	26(86.7%)	11(84.6%)	24(85.7%)	186(87.7%)	12(80.0%)	277(84.5%)
Artemether / Lumefantrine 20/120 mg	5(83.3%)	20(83.3%)	29(96.7%)	11(84.6%)	26(92.9%)	167(78.8%)	14(93.3%)	272(82.9%)
Metronidazole Suspension 100 mg/5 ml	5(83.3%)	11(45.8%)	25(83.3%)	10(76.9%)	20(71.4%)	175(82.5%)	12(80.0%)	268(78.7%)
Ampicillin 125 mg + Cloxacillin 125 mg/5 ml	6(100.0%)	14(58.3%)	27(90.0%)	11(84.6%)	21(75.0%)	175(82.5%)	13(86.7%)	267(81.4%)
Ampicillin 250 mg + Cloxacillin 250 mg	6(100.0%)	15(62.5%)	27(90.0%)	9(69.2%)	24(85.7%)	174(82.1%)	8(53.3%)	263(80.2%)
Cetrizine Syrup 1 mg/Ml	6(100.0%)	17(70.8%)	25(83.3%)	9(69.2%)	20(71.4%)	171(80.7%)	14(93.3%)	262(79.9%)
Dextrose 5% solution	3(50.0%)	3(12.5%)	3(10.0%)	4(30.8%)	4(14.3%)	44(20.8%)	1(6.7%)	62(18.9%)
ORS	3(50.0%)	2(8.3%)	5(16.7%)	3(23.1%)	5(17.9%)	41(19.3%)	3(20.0%)	62(18.9%)
Sodium Chloride 0.9% solution	2(33.3%)	6(25.0%)	6(20.0%)	4(30.8%)	4(14.3%)	35(16.5%)	1(6.7%)	58(17.7%)
Dextrose 50% solution	0(0.0%)	2(8.3%)	7(23.3%)	3(23.1%)	2(7.1%)	34(16.0%)	9(60.0%)	57(17.4%)
Hartmann’s Ringers Lactate solution	2(33.3%)	3(12.5%)	3(10.0%)	4(30.8%)	4(14.3%)	33(15.6%)	0(0.0%)	49(14.9%)
Zinc Solution Supplement 10 mg/5 ml	0(0.0%)	0(0.0%)	0(0.0%)	0(0.0%)	3(10.7%)	11(5.2%)	0(0.0%)	14(4.3%)
Surgical Spirit	0(0.0%)	0(0.0%)	0(0.0%)	0(0.0%)	1(3.6%)	5(2.4%)	0(0.0%)	6(1.8%)
ORS + Zinc Sulphate Monohydrate 20 mg Tablets	0(0.0%)	0(0.0%)	0(0.0%)	0(0.0%)	0(0.0%)	1(0.5%)	0(0.0%)	1(0.3%)

### Impact of the import verification fees increment on the cost of the selected essential medicines

#### Median unit prices before and after the increment in import verification fees

The adjusted median prices of both local and imported medicines generally increased or remained the same after the introduction of import verification fees. The prices of 8 (19.0%) of the local and 7 (16.7%) of the imported medicines remained the same. The increase in price of local products was only significant for ciprofloxacin 500 mg (P = 0.041) while for the imported products, the increase was significant for Artemether / Lumefantrine 15/90 Dry Suspension (P = 0.009), Quinine Sulphate 300 mg Tablets (P = 0.021), surgical spirit (P = 0.0003) and Zinc Sulfate Monohydrate BP (54.90) Equivalent To 20 mg Elemental Zinc (P = 0.025). The median prices of local products were generally lower than the imported products. Details in Table 8 and table S3.

**Table 8:** Comparison of unit median prices after adjusting for inflation/deflation

Brands	Local			Imported		
	Median price Before	Current median price	*P*-Value	Median price Before	Current median price	*P*-Value
Albendazole Suspension 100 mg/5 ml	29.7	29.7	1.00	107.3	115.5	0.50
Amoxicillin Trihydrate Equivalent To Amoxicillin 250 mg	33.0	45.4	0.50	33.0	33.0	1.00
Ampicillin 250 mg + Cloxacillin 250 mg	53.7	66.0	0.206	66.0	66.0	1.00
Artemether / Lumefantrine 15/90 Dry Suspension	-	-	-	57.6	68.7	**0.009**
Cetirizine Hydrochloride 10 mg Tablets	16.5	33.0	0.50	66.0	66.0	1.00
Cetrizine Syrup 1 mg/Ml	21.8	21.8	1.00	27.4	30.2	0.50
Ciprofloxacin 500 mg	31.6	49.5	**0.041**	445.5	528.0	0.50
Hartmann’s Ringers Lactate solution	660	660	1.00	660	825	0.50
Ibuprofen 200 mg Tablet	16.5	16.5	1.00	16.5	24.8	0.50
Loperamide 2 mg	33	33	1.00	33	74.3	0.50
Mannitol 20%	1320	1122	0.50	1386	1386	1.00
Metronidazole 200 mg Tablets /	14.3	14.5	0.50	16.5	16.5	1.00
Sodium Chloride 0.9% solution	660	825	0.50	660	660	1.00
ORS	165	165	1.00	145.2	148.5	0.50
Paracetamol 500 mg Tablets / Suspension 120 mg/5 ml	12.4	12.4	1.00	43.8	49.5	0.256
Quinine Sulphate 300 mg Tablets	66.0	90.8	0.205	70.2	95.7	**0.021**
Surgical Spirit	511.5	561	0.50	325.9	82.5	**0.0003**
Sulfamethoxazole 400 mg / Trimethoprim 80 mg	22.3	24.0	0.50	33	33	1.00
Zinc Sulfate Monohydrate BP (54.90) Equivalent To 20 mg Elemental Zinc	33	33	1.00	33	66	**0.025**

#### Comparison of unit median prices after adjusting for inflation/deflation for cheapest priced medicines

The median prices of both local and imported lowest priced medicines also generally increased or remained the same after the introduction of import verification fees. The prices of 16 (36.4%) of the local and 13 (29.5%) of the imported medicines remained the same. The increase in price of local products was significant for 10 (23.8%) of the products while for the imported products, the increase was significant for 7 (15.9%) of the products. The median prices of local products were generally lower than the imported products. Details in Table 9 and table S4.

**Table 9:** Comparison of median prices of lowest priced imported and locally manufactured medicines

Brands	Local			Imported		
	Median price before	Current median price	*P*-value	Median price before	Current median price	*P*-Value
Albendazole 400 mg Tablet	330	330	1	660	775.5	**0.001**
Albendazole Suspension 100 mg/5 ml	29.7	29.7	1	82.5	99	0.165
Amoxicillin Trihydrate Equivalent To Amoxicillin 250 mg	33	33	1	33	33	1
Ampicillin 250 mg + Cloxacillin 250 mg	57.75	66	0.25	66	66	1
Artemether / Lumefantrine 20/120 mg	49.5	68.64	**0.034**	61.38	68.64	0.26
Artemether / Lumefantrine 15/90 Dry Suspension	0	0	1	60.39	71.28	0.189
Ascorbic Acid (Vitamin C 100 mg) Tablet	16.5	33	**0.026**	16.5	33	0.165
Cetirizine Hydrochloride 10 mg Tablets	16.5	33	**0.046**	66	66	1
Cetrizine Syrup 1 mg/Ml	21.78	21.78	1	27.39	33	0.261
Ciprofloxacin 500 mg	49.5	66	0.165	66	66	1
Ciprofloxacin 0.2% solution	495	660	**0.016**	660	825	**0.017**
Dextrose 5% solution	660	825	**0.016**	660	825	**0.017**
Dextrose 50% solution	1105.5	1155	**0.023**	1320	1650	**0.003**
Erythromycin 250 mg tab	44.55	66	**0.035**	66	66	1
Hartmann’s Ringers Lactate solution	660	660	1	660	825	**0.017**
Ibuprofen 200 mg Tablet	16.5	16.5	1	16.5	16.5	1
Ibuprofen Suspension 100 mg/5 ml	6.6	6.6	1	13.2	16.5	0.33
Loperamide 2 mg	33	33	1	33	33	1
Mannitol 20%	1320	1320	1	1320	1650	**0.003**
Metronidazole 0.5%	495	660	**0.016**	660	660	1
Metronidazole 200 mg Tablets /	16.5	16.5	1	16.5	16.5	1
Sodium Chloride 0.9% solution	660	825	**0.017**	660	660	1
Omeprazole 20 mg	33	0	1	49.5	66	0.65
ORS	165	165	1	165	165	1
ORS + Zinc Sulphate Monohydrate 20 mg Tablets	990	990	1	0	0	1
Paracetamol 500 mg Tablets	16.5	16.5	1	29.7	33	0.33
Surgical Spirit	528	627	**0.02**	379.5	99	**0.003**
Sulfamethoxazole 400 mg / Trimethoprim 80 mg	33	33	1	33	33	1
Zinc Sulfate Monohydrate BP (54.90) Equivalent To 20 mg Elemental Zinc	33	33	1	33	66	0.063

#### Median price ratios

The MPR for most of the medicines were within the acceptable range of 3 or less for both local and imported medicines. The medicines with high MPR before remained high. However, the number of medicines with MPR (1 or more) were more currently compared to before introduction of verification fees for both imported and locally produced medicines. In 2020, the median unit prices of 12 (28.6%) locally produced medicines and 20 (47.6%) imported medicines were higher than the international median unit prices. Details in Table 10 and table S5.

**Table 10:** International comparison of median unit prices

Brand names	Median price/ Local 2017	MPR	Median price/ Local 2020	MPR	Median price/ Imported 2017	MPR	Median price/ Imported 2020	MPR
Albendazole 400 mg Tablet	198	3.8	288.75	5.5	742.5	14.2	940.5	18.0
Albendazole Suspension 100 mg/5 ml	29.7	0.9	29.7	0.9	107.3	3.2	115.5	3.5
Ampicillin 125 mg + Cloxacillin 125 mg/5 ml	8.7	0.5	29.4	1.6	15.7	0.9	16.5	0.9
Ampicillin 250 mg + Cloxacillin 250 mg	53.7	0.8	66	1.0	66	1.0	66	1.0
Ascorbic Acid (Vitamin C 100 mg) Tablet	16.5	0.7	37.2	1.5	16.5	0.7	33	1.3
Cetirizine Hydrochloride 10 mg Tablets	16.5	0.5	33	1.0	66	2.0	66	2.0
Cetrizine Syrup 1 mg/Ml	21.8	1.2	21.8	1.2	27.4	1.6	30.2	1.7
Ciprofloxacin 500 mg	31.6	0.2	49.5	0.4	445.5	3.3	528	3.9
Ciprofloxacin 0.2% solution	495	1.9	660	2.5	643.5	2.4	783.8	3.0
Dextrose 5% solution	660	20.0	825	25.0	660	20.0	825	25.0
Dextrose 50% solution	1105.5	20.6	1155	21.6	1485	27.7	1650	30.8
Diclofenac Sodium 100 mg	13.1	0.0	21.6	0.0	528	1.1	627	1.3
Doxycycline 100 mg	33	0.7	49.5	1.0	74.3	1.5	82.5	1.7
Hartmann’s Ringers Lactate solution	660	179.8	660	179.8	660	179.8	825	224.8
Ibuprofen 200 mg Tablet	16.5	0.7	16.5	0.7	16.5	0.7	24.8	1.0
Loperamide 2 mg	33	1.0	33	1.0	33	1.0	74.3	2.2
Mannitol 20%	1320	35.6	1122	30.3	1386	37.4	1386	37.4
Metronidazole 0.5%	495	27.0	660	36.0	577.5	31.5	660	36.0
Sodium Chloride 0.9% solution	660	179.8	825	224.8	660	179.8	660	179.8
Omeprazole 20 mg	33	0.7	-		57.8	1.2	66	1.4
Paracetamol 500 mg Tablets	12.4	0.8	12.4	0.8	43.8	2.7	49.5	3.1
Paracetamol Suspension 120 mg/5 ml	5.5	0.3	6.6	0.3	18.2	1.0	19.8	1.0
Sulfamethoxazole 200 mg / Trimethoprim 400 mg / 5 ml	6.2	0.4	10.6	0.7	13.4	0.9	15.9	1.0
Surgical Spirit	511.5	25.8	561	28.3	325.9	16.4	82.5	4.2
Zinc Sulfate Monohydrate BP (54.90) Equivalent To 20 mg Elemental Zinc	33	0.8	33	0.8	33	0.8	66	1.6
Zinc Solution Supplement 10 mg/5 ml	13.5	0.9	10.6	0.7	14.2	0.9	16.5	1.0

#### Key informant perspectives on the price and availability of the 37 selected essential medicines after increase in import verification fees

The price of the selected locally manufactured medicines did not reduce after the increment in verification fees according to the key informants. They however noted that the prices for these products remained competitive in the market and are favorable to the consumers.

On the other hand, some key informants reported no reduction and increase in prices not only for the selected products but also other products citing reasons of increment in verification fees for the increase.

The policy led to increase in prices to patients and the increment did not stop at the 37 products only, it was instead used as an opportunity by importers to increase even other products because they had reasons of tax increment to give to customers KI05.

*“Despite the increase in the levy from 2–12%, imported commodities are still less costly compared to the locally manufactured commodities. I do not have examples in my mind but we have items, which are actually overpriced compared to the imported items of the same KI07”.*

Generally, the key informants reported no increase in availability of the selected products. Some of the key informants instead noted shortages of the selected medicines as a result of lack of capacity of local manufacturers to satisfy the local market, source of raw materials from other countries and failure to process and deliver orders timely. It was reported that the high cost of importation discouraged importation of some of the products which affects availability of the products.

*“The increment in verification fees increased the efforts of local manufacturers towards making the capped products available in the market all the time in order to satisfy the needs of local people KI03”.*

“*The increment in verification has led to shortages of drugs experienced in Uganda because the local manufacturers lack capacity to satisfy the local market demand. They source raw materials from other countries hence affecting availability of drugs and production capacity KI01”.*

*“Some of the capped products are always in short supply. Sometimes our orders are not delivered on time, instead we are put on schedule that makes us wait for weeks or even months before the products are delivered. This shortage of products has in turn led to increase in prices which are felt by the end users of these products K104”.*

We had an assumption that we would get these products cheaper than imported ones but it has not come to pass. It is the end user who is suffering, paying high costs hence affecting availability K106.

#### Challenges faced by local manufacturers based according to the key informants

The key informants reported a number of challenges that hindered achievement of the objectives of the increment in import verification fees. These included;

1. Provision of export subsidies by countries from which the medicines are imported into the country. This according to the key informants reduces the competitive power of local manufacturers in the market. It was noted that even at 12% verification fees, some importers were still importing some of the 37 capped products cheaply where they get export subsidies.
2. Presence of companies in the market who are manufacturing and importing at the same time. This according to the key informants has led to monopolistic tendencies where few players with high volumes control the market. This in their view creates conflict of interest and price manipulations since these players manipulate prices without interference.
3. The high cost of imported raw materials increase prices of the items higher than those imported from other countries.
4. Lack of price control in the market was also mentioned as a challenge.

*Recommendations for improving the effectiveness of the policy in increasing access to medicines from the key informants*.

i. The policy should be extended to include all the essential medicines.
ii. The verification fees should be increased to 16 − 25% to deter importation.
iii. Local manufacturers should be given complete exclusivity to produce certain products with no importation.

## Discussion of results

The findings of this study provide evidence on the availability and the price of selected medicines after the increment of import verification fees on availability and cost of medicines. This evidence is important as the country seeks to reduce over dependence on imports and international donations but at the same time promote access to affordable essential medicines. The few countries (13%) that levied tariffs on imported finished products between 10 and 20% unlike Uganda are in the middle income bracket and had the capacity of locally producing medicines in quantities that can satisfy the country’s demand as revealed by a previous study [7]. This current study reveals the impact of such high tariffs for a low-income country developing capacity to adequately satisfy its local demand.

### Impact of the import verification fees increment on the availability of essential medicines

The main objective of a national medicine policy is to ensure the availability, affordability, accessibility and rational use of essential medicines that are safe, effective and quality assured. As one of the means to achieving this, governments in LMICs support local medicine production, expecting it to result in increased availability and lower prices for medicines in addition to industrial and economic benefits [14]. A few studies have highlighted the impact of local production on availability and prices of medicines unlike the general information on medicine availability and prices where extensive literature exists.

In the current study, the overall availability of imported medicines (54.8%) was higher than the local medicines (37.1%). A study comparing the price and availability of locally produced and imported medicines in Tanzania and Ethiopia found a similar pattern in Tanzania but the reverse in Ethiopia [15]. In Tanzania, it was found that availability of local and imported products was 21% and 70%, respectively. From the same study, the availability of local and imported products was 54% and 35%, respectively in Ethiopia. Unlike this study, the study conducted in Tanzania and Ethiopia assessed availability in both private and public sector.

Locally produced parenteral preparations (54.6%) were the only formulations more available than imported products. This could be due to the fact that Uganda has a local manufacturing facility specifically for production of parenteral products and the ability of the industry to meet the market demand and their wide spread distribution network.

The availability of local brands was mainly low (45%) and very low (41%) while the imported brands were mainly fairly high (74%). Generally, 65% of the brands available in the surveyed pharmacies were imported. This means that the imported products still dominate the market after the imposition of the verification fees. This is in agreement with previous literature which suggests that the Ugandan market is dominated by imports which constituted close to 90% [16] of Essential Medicines and Health Supplies in the country [17]. On the other hand, it also indicates a reduction in dominance of the Ugandan market with the imports of the selected medicines and improved availability of locally produced medicines since the dominance has reduced to 65%. However, a better indication of improvement or no improvement in availability would have been shown by changes in availability before and after the introduction of import verification fees. This was not possible in this study because of inadequacies in record keeping.

Very few local (4.7%) and imported (25.6%) medicines met WHO global action plan target of 80% availability of essential medicines by 2025 in public and private sectors. A baseline assessment of WHO’s target for availability of essential medicines revealed 18.9% and 5.2% availability of the lowest-priced generics and originator brands respectively that met WHO’s target in private sector in low income countries [18]. Although local and imported medicines may not be comparable to lowest priced and originator brands, the lowest priced generics in this study can be compared to the local medicines since most of them had the lowest prices. Therefore, the availability of local medicines was much lower compared to the findings of the baseline assessment study. A similar trend was reported by a study conducted in Pakistan that suggested poor availability of lowest priced generics (20.3%) and originator brands (55.0%) in private sector facilities [19].

The key informants reported no increase in availability of the selected products. Some of the reasons noted by key informants contributing to this picture included lack of capacity of local manufacturers to satisfy the local market, sourcing of raw materials from other countries and failure to process and deliver orders timely. It was reported by key informants that the high cost of importation discouraged importation of some of the products which affects availability of the products.

### Impact of the import verification fees increment on the cost of the affected 37 selected essential medicines

Tariffs such as import verification fees are vital determinants of prices and they can considerably increase the prices of imported goods [7]. According to WHO, taxes account for 20 to 30% of the final price patients pay for medicines [19]. A number of countries have adopted removing or reducing taxes and tariffs on medicines as a strategy of realizing a decrement in the retail cost and subsequent affordability and availability of medicines to the final consumer [20, 21]. The Ugandan government’s initiative to increase import verification taxes on 37 selected medicines from 2 to 12% while leaving locally manufactured products at fees of 2% was expected to increase the prices of imported medicines giving comparative price advantage to the locally produced products. However, the median prices of both local and imported medicines generally increased or remained the same after the introduction of import verification fees. The increase in median price of local products was significant for only one product while for the imported products, the increase was significant for four (4) products and the median prices of local products were generally lower than the imported products. Considering lowest priced medicines, the prices of 16 (36.4%) of the local and 13 (29.5%) of the imported medicines remained the same and the increase in price of local products was significant for 9 (20.5%) of the products while for the imported products, the increase was significant for 7 (15.9%) of the products. Whereas it makes logical sense to expect decrease or no increase in cost of locally produced medicines accruing from economies of scale and price advantage, the increase in verification fees has not resulted in reduction in prices of locally produced medicines. This was also noted by most of the key informants who reported that, the prices of the selected locally manufactured medicines remained the same or increased after the increment in verification fees but remained competitive in the market. Similarly, in Peru, removal of indirect taxes on a range of cancer medicines and antiretroviral medicines resulted in little reduction in prices [22].

As per the findings and observation of the key informants, the prices of medicines were within acceptable international prices. The MPR for most of the medicines were within the acceptable range of 3 or less of private sector for both local and imported medicines. WHO considers patient prices to be high when MPRs exceed four (4). The medicines with high MPR before remained high. However, the number of medicines with MPR (1 or more) were more in 2020 compared to before introduction of verification fees in 2017 for both imported and local brands. In 2020, the median unit prices of 12 (28.6%) locally produced medicines and 20 (47.6%) imported medicines were higher than the international median unit prices compared to 11 (25.0%) locally produced medicines and 20 (45.5%) imported medicines before introduction of import verification fees. This is an indication of increase in prices of medicines, high prices paid by patients to access these medicines and imported medicines having higher median prices. The study comparing the price and availability of locally produced and imported medicines in Tanzania and Ethiopia found a similar pattern of higher median price ratios for imported medicines compared to local produced medicines [15]. From the study, patient prices for local products (median MPR = 1.85) were lower than imported products (median MPR = 5.42) in Ethiopia but almost identical in Tanzania (Median MPR = 2.27 for locally produced products and Median MPR = 2.29 for imported products). However, this study could not establish whether these prices are a reflection of manufacturer’s selling prices. It is possible that local manufacturers may be selling at lower prices but add-ons and manipulation of prices by distributors increase the prices making the medicines expensive for the patients. This study didn’t assess the medicine price components such as manufacturer’s selling prices, mark-ups and other add-ons in the supply chain that make up the final patient price.

It is therefore important as governments support local production, it should as well assess price components and then regulate the markets to ensure their support results in more affordable medicines for patients [23]. For example South Africa adopted a Single Exit Price (SEP) mechanism that bans discounts and rebates and provides transparent information about the prices of medicines sold in the private sector [24].

The increase or no change in prices for local products was attributed to export subsidies from the countries where products are imported reducing the competitive power of local manufacturers in the market, presence of local manufacturers who are importing at the same time, high cost of raw materials and lack of price control in the market. It was noted that even at 12% verification fees, some importers were still importing some of the 37 capped products from some countries cheaply where they are given export subsidies. According to the key informants, presence of local manufacturers who also import medicines led to monopolistic tendencies where few players with high volumes control the market and manipulate prices without interference. The increase in prices for imported products was attributed to increase in verification fees.

High prices of medicines reduce utilization particularly by the poor and elderly, and reduce compliance with preventive and chronic disease treatment regimens leading to poor health outcomes. Therefore, in promoting local production, dual policy objectives need to be explored. In the short to medium term, governments need to develop and implement policies through which they can continue to support local production but, at the same time, prevent high prices being passed on to patients.

### Study limitations

Because of poor records, only point availability was determined therefore comparison of availability of medicines before introduction of the verification fees was not established. The assessment was limited to the private sector; public sector pharmacies were not included. However, since all government facilities are supplied by only one supplier, the prices of medicines in public pharmacies would be uniform throughout Uganda and the perspectives of key informants from public institutions were sought. It is possible that COVID-19 pandemic might have had an impact on the findings but shouldn’t have been significant since most of the time period 2017, 2018 and 2019 were not affected by the pandemic and supply chain activities continued throughout COVID-19 pandemic.

## Conclusions

The overall availability of imported medicines was higher than the local medicines. The availability varied across all the regions. Locally produced parenteral preparations were the only formulations more available than imported products. The availability of local brands was mainly low and very low while the imported brands were mainly fairly high. Most of the brands available in the surveyed pharmacies were imported.

The median prices of imported medicines were generally higher than local products and most of the medicines had an acceptable MPR of 3 or less for private sector. The median prices of both local and imported medicines generally increased or remained the same after the introduction of import verification fees.

### Over all recommendations

1. Local manufacturers should demonstrate capacity to produce a given product before they are added on the list of capped products.
2. There is a need for price controls and transparency in the private sector.

### Future studies

1. A study to assess the medicine price components such as manufacturer’s selling prices, mark-ups and other add-ons in the supply chain that make up the final patient price should be conducted.
2. A study on production volumes to adequately meet the demands of the country and the capacity of the industry to produce the quantities should be conducted.

### Electronic supplementary material

Below is the link to the electronic supplementary material.

Supplementary Material 1

## Data Availability

The data for the study are available from the corresponding author upon reasonable request.

## Abbreviations

ANOVA: Analysis of Variance
BUBU: Buy Uganda Build Uganda
CEHURD: Center for Health, Human Rights and Development
COVID: Corona virus Disease
CPI: Consumer Price Index
MOH: Ministry of Health
MPR: Median Price Ratio
MSH: Management Sciences for Health
NDA: National Drug Authority
NDP: National Drug Policy
PI: Principal Investigator
UBOS: Uganda Bureau of Statistics
UPOA: Uganda Pharmacy owners association
VEN: Vital Essential and Necessary
WHO: World Health Organization
