# Supplementary material for: Cost and availability of selected medicines after implementation of increased import verification fees
Source: BMC Health Serv Res. 2024 Jan 4;24:25. doi: 10.1186/s12913-023-10433-7 (PMC10768383; doi:10.1186/s12913-023-10433-7)
Supplement: Supplementary file 1 — Supplementary Material 1 [file 12913_2023_10433_MOESM1_ESM.doc]

Supplementary results

Supplementary Materials: Table S1: Individual local medicine availability by region and overall availability in all regions, Table S2: Individual imported medicine availability by region and overall availability in all regions; Table S3: Crude Median unit prices before and after the policy for local and imported products; Table S4: Comparison of unit median prices after adjusting for inflation/deflation; Table S5 comparison of median prices of lowest priced imported and local produced medicines; Table S6: International comparison of median unit prices; Table S7: Mean availability of local manufactured medicines; Table S8 : Mean Availability of imported medicines; Table S9 : Impact of 12% increase in import verification fees on price of medicines : Table S10; Impact of 12% increase in import verification fees on availability of drugs; S11: Key informant guide

Table S1: Individual local medicine availability by region and overall availability in all regions

| Brand Names | Arua (n=6) | Hoima (n=24) | Jinja (n=30) | Lira (n=13) | Mbarara (n=28) | Nakawa (n=212) | Tororo (n=15) | Overall (n=328) |
| --- | --- | --- | --- | --- | --- | --- | --- | --- |
| Albendazole 400mg Tablet | 3(50.0%) | 10(41.7%) | 10(33.3%) | 1(7.7%) | 6(21.4%) | 69(32.5%) | 9(60.0%) | 108(32.9%) |
| Albendazole Suspension 100mg/5ml | 0(0.0%) | 0(0.0%) | 0(0.0%) | 0(0.0%) | 1(3.6%) | 3(1.4%) | 0(0.0%) | 4(1.2%) |
| Amoxicillin Trihydrate Equivalent To Amoxicillin 125mg/5ml | 0(0.0%) | 8(33.3%) | 4(13.3%) | 2(15.4%) | 4(14.3%) | 25(11.8%) | 3(20.0%) | 46(14.0%) |
| Amoxicillin Trihydrate Equivalent To Amoxicillin 250mg | 2(33.3%) | 10(41.7%) | 11(36.7%) | 5(38.5%) | 9(32.1%) | 68(32.1%) | 4(26.7%) | 109(33.2%) |
| Ampicillin 125mg + Cloxacillin 125mg/5ml | 0(0.0%) | 12(50.0%) | 4(13.3%) | 1(7.7%) | 5(17.9%) | 31(14.6%) | 5(33.3%) | 58(17.7%) |
| Ampicillin 250mg + Cloxacillin 250mg | 0(0.0%) | 9(37.5%) | 6(20.0%) | 5(38.5%) | 3(10.7%) | 53(25.0%) | 8(53.3%) | 84(25.6%) |
| Artemether / Lumefantrine 20/120mg | 1(16.7%) | 4(16.7%) | 3(10.0%) | 3(23.1%) | 3(10.7%) | 81(38.2%) | 0(0.0%) | 95(29.0%) |
| Artemether / Lumefantrine 15/90 Dry Suspension | 0(0.0%) | 0(0.0%) | 2(6.7%) | 0(0.0%) | 0(0.0%) | 1(0.5%) | 0(0.0%) | 3(0.9%) |
| Ascorbic Acid (Vitamin C 100mg) Tablet | 1(16.7%) | 18(75.0%) | 19(63.3%) | 10(76.9%) | 17(60.7%) | 124(58.5%) | 14(93.3%) | 203(61.9%) |
| Cetirizine Hydrochloride 10mg Tablets | 4(66.7%) | 20(83.3%) | 22(73.3%) | 12(92.3%) | 23(82.1%) | 102(48.1%) | 14(93.3%) | 197(60.1%) |
| Cetrizine Syrup 1mg/Ml | 0(0.0%) | 3(12.5%) | 0(0.0%) | 0(0.0%) | 0(0.0%) | 7(3.3%) | 0(0.0%) | 10(3.0%) |
| Chloramphenicol Palmitate 125/5ml | 2(33.3%) | 8(33.3%) | 2(6.7%) | 5(38.5%) | 7(25.0%) | 37(17.5%) | 7(46.7%) | 68(20.7%) |
| Ciprofloxacin 500mg | 3(50.0%) | 5(20.8%) | 2(6.7%) | 1(7.7%) | 2(7.1%) | 30(14.2%) | 4(26.7%) | 47(14.3%) |
| Ciprofloxacin 0.2% solution | 0(0.0%) | 9(37.5%) | 15(50.0%) | 2(15.4%) | 9(32.1%) | 47(22.2%) | 2(13.3%) | 84(25.6%) |
| Cloxacillin 125mg/5ml | 0(0.0%) | 1(4.2%) | 1(3.3%) | 0(0.0%) | 3(10.7%) | 7(3.3%) | 1(6.7%) | 13(4.0%) |
| Cloxacillin Sodium Equivalent To Cloxacillin 250mg | 3(50.0%) | 9(37.5%) | 7(23.3%) | 6(46.2%) | 5(17.9%) | 58(27.4%) | 4(26.7%) | 92(28.0%) |
| Dextrose 5% solution | 3(50.0%) | 20(83.3%) | 27(90.0%) | 8(61.5%) | 23(82.1%) | 150(70.8%) | 13(86.7%) | 244(74.4%) |
| Dextrose 50% solution | 3(50.0%) | 14(58.3%) | 20(66.7%) | 7(53.8%) | 22(78.6%) | 118(55.7%) | 6(40.0%) | 190(57.9%) |
| Diclofenac Sodium 100mg | 0(0.0%) | 2(8.3%) | 1(3.3%) | 0(0.0%) | 6(21.4%) | 17(8.0%) | 5(33.3%) | 31(9.5%) |
| Doxycycline 100mg | 3(50.0%) | 17(70.8%) | 19(63.3%) | 5(38.5%) | 15(53.6%) | 119(56.1%) | 10(66.7%) | 188(57.3%) |
| Erythromycin 125mg/5ml | 4(66.7%) | 15(62.5%) | 8(26.7%) | 3(23.1%) | 11(39.3%) | 42(19.8%) | 6(40.0%) | 89(27.1%) |
| Erythromycin 250mg tab | 0(0.0%) | 6(25.0%) | 5(16.7%) | 4(30.8%) | 7(25.0%) | 55(25.9%) | 10(66.7%) | 87(26.5%) |
| Hartmann’s Ringers Lactate solution | 4(66.7%) | 20(83.3%) | 25(83.3%) | 7(53.8%) | 22(78.6%) | 128(60.4%) | 13(86.7%) | 219(66.8%) |
| Ibuprofen 200mg Tablet | 2(33.3%) | 14(58.3%) | 18(60.0%) | 7(53.8%) | 15(53.6%) | 79(37.3%) | 10(66.7%) | 145(44.2%) |
| Ibuprofen Suspension 100mg/5ml | 2(33.3%) | 8(33.3%) | 8(26.7%) | 5(38.5%) | 6(21.4%) | 30(14.2%) | 5(33.3%) | 64(19.5%) |
| Loperamide 2mg | 3(50.0%) | 15(62.5%) | 24(80.0%) | 9(69.2%) | 19(67.9%) | 126(59.4%) | 12(80.0%) | 208(63.4%) |
| Magnesium Trisilicate 250+Dried Aluminium Hydroxide 120/Gel | 1(16.7%) | 3(12.5%) | 6(20.0%) | 9(69.2%) | 6(21.4%) | 50(23.6%) | 9(60.0%) | 84(25.6%) |
| Mannitol 20% | 0(0.0%) | 5(20.8%) | 13(43.3%) | 2(15.4%) | 14(50.0%) | 29(13.7%) | 3(20.0%) | 66(20.1%) |
| Metronidazole 0.5% | 1(16.7%) | 6(25.0%) | 16(53.3%) | 5(38.5%) | 16(57.1%) | 49(23.1%) | 3(20.0%) | 96(29.3%) |
| Metronidazole 200mg Tablets / | 2(33.3%) | 8(33.3%) | 13(43.3%) | 8(61.5%) | 4(14.3%) | 62(29.2%) | 7(46.7%) | 104(31.7%) |
| Metronidazole Suspension 100mg/5ml | 1(16.7%) | 9(37.5%) | 7(23.3%) | 2(15.4%) | 2(7.1%) | 40(18.9%) | 2(13.3%) | 63(19.2%) |
| Sodium Chloride 0.9% solution | 4(66.7%) | 19(79.2%) | 24(80.0%) | 9(69.2%) | 24(85.7%) | 146(68.9%) | 13(86.7%) | 239(72.9%) |
| Omeprazole 20mg | 0(0.0%) | 1(4.2%) | 1(3.3%) | 0(0.0%) | 0(0.0%) | 1(0.5%) | 0(0.0%) | 3(0.9%) |
| ORS | 5(83.3%) | 20(83.3%) | 26(86.7%) | 10(76.9%) | 22(78.6%) | 187(88.2%) | 12(80.0%) | 282(86.0%) |
| ORS + Zinc Sulphate Monohydrate 20mg Tablets | 0(0.0%) | 1(4.2%) | 5(16.7%) | 2(15.4%) | 1(3.6%) | 23(10.8%) | 4(26.7%) | 36(11.0%) |
| Paracetamol 500mg Tablets | 4(66.7%) | 22(91.7%) | 24(80.0%) | 9(69.2%) | 27(96.4%) | 143(67.5%) | 13(86.7%) | 242(73.8%) |
| Paracetamol Suspension 120mg/5ml | 0(0.0%) | 8(33.3%) | 5(16.7%) | 0(0.0%) | 1(3.6%) | 19(9.0%) | 0(0.0%) | 33(10.1%) |
| Quinine Sulphate 300mg Tablets / Syrup 100mg/5ml | 2(33.3%) | 10(41.7%) | 14(46.7%) | 9(69.2%) | 11(39.3%) | 96(45.3%) | 11(73.3%) | 153(46.6%) |
| Quinine Sulphate Syrup 100mg/5ml | 3(50.0%) | 17(70.8%) | 11(36.7%) | 6(46.2%) | 10(35.7%) | 75(35.4%) | 8(53.3%) | 130(39.6%) |
| Sulfamethoxazole 200mg / Trimethoprim 400mg / 5ml | 1(16.7%) | 10(41.7%) | 6(20.0%) | 3(23.1%) | 6(21.4%) | 27(12.7%) | 8(53.3%) | 61(18.6%) |
| Surgical Spirit | 5(83.3%) | 20(83.3%) | 26(86.7%) | 12(92.3%) | 25(89.3%) | 192(90.6%) | 15(100.0%) | 295(89.9%) |
| Sulfamethoxazole 400mg / Trimethoprim 80mg | 2(33.3%) | 18(75.0%) | 20(66.7%) | 5(38.5%) | 12(42.9%) | 88(41.5%) | 9(60.0%) | 154(47.0%) |
| Zinc Sulfate Monohydrate BP (54.90) Equivalent To 20mg Elemental Zinc | 2(33.3%) | 13(54.2%) | 3(10.0%) | 3(23.1%) | 2(7.1%) | 63(29.7%) | 8(53.3%) | 94(28.7%) |
| Zinc Solution Supplement 10mg/5ml | 3(50.0%) | 9(37.5%) | 16(53.3%) | 7(53.8%) | 11(39.3%) | 82(38.7%) | 8(53.3%) | 136(41.5%) |

Individual selected local medicine availability by region and overall availability in all regions

| Brand Names | Arua | Hoima | Jinja | Lira | Mbarara | Nakawa | Tororo | Overall |
| --- | --- | --- | --- | --- | --- | --- | --- | --- |
| Albendazole Suspension 100mg/5ml | 0(0.0%) | 0(0.0%) | 0(0.0%) | 0(0.0%) | 1(3.6%) | 3(1.4%) | 0(0.0%) | 4(1.2%) |
| Artemether / Lumefantrine 15/90 Dry Suspension | 0(0.0%) | 0(0.0%) | 2(6.7%) | 0(0.0%) | 0(0.0%) | 1(0.5%) | 0(0.0%) | 3(0.9%) |
| Cetrizine Syrup 1mg/Ml | 0(0.0%) | 3(12.5%) | 0(0.0%) | 0(0.0%) | 0(0.0%) | 7(3.3%) | 0(0.0%) | 10(3.0%) |
| Cloxacillin 125mg/5ml | 0(0.0%) | 1(4.2%) | 1(3.3%) | 0(0.0%) | 3(10.7%) | 7(3.3%) | 1(6.7%) | 13(4.0%) |
| Dextrose 5% solution | 3(50.0%) | 20(83.3%) | 27(90.0%) | 8(61.5%) | 23(82.1%) | 150(70.8%) | 13(86.7%) | 244(74.4%) |
| Hartmann’s Ringers Lactate solution | 4(66.7%) | 20(83.3%) | 25(83.3%) | 7(53.8%) | 22(78.6%) | 128(60.4%) | 13(86.7%) | 219(66.8%) |
| Sodium Chloride 0.9% solution | 4(66.7%) | 19(79.2%) | 24(80.0%) | 9(69.2%) | 24(85.7%) | 146(68.9%) | 13(86.7%) | 239(72.9%) |
| ORS | 5(83.3%) | 20(83.3%) | 26(86.7%) | 10(76.9%) | 22(78.6%) | 187(88.2%) | 12(80.0%) | 282(86.0%) |
| Paracetamol 500mg Tablets | 4(66.7%) | 22(91.7%) | 24(80.0%) | 9(69.2%) | 27(96.4%) | 143(67.5%) | 13(86.7%) | 242(73.8%) |
| Omeprazole 20mg | 0(0.0%) | 1(4.2%) | 1(3.3%) | 0(0.0%) | 0(0.0%) | 1(0.5%) | 0(0.0%) | 3(0.9%) |

Table S2: Individual imported medicine availability by region and overall availability in all regions

| Brands | Arua (n=6) | Hoima (n=24) | Jinja (n=30) | Lira (n=13) | Mbarara (n=28) | Nakawa (n=212) | Tororo (n=15) | Overall (n=328) |
| --- | --- | --- | --- | --- | --- | --- | --- | --- |
| Albendazole 400mg Tablet | 3(50.0%) | 15(62.5%) | 26(86.7%) | 11(84.6%) | 24(85.7%) | 186(87.7%) | 12(80.0%) | 277(84.5%) |
| Albendazole Suspension 100mg/5ml | 0(0.0%) | 7(29.2%) | 25(83.3%) | 12(92.3%) | 15(53.6%) | 173(81.6%) | 14(93.3%) | 246(75.0%) |
| Amoxicillin Trihydrate Equivalent To Amoxicillin 125mg/5ml | 6(100.0%) | 20(83.3%) | 29(96.7%) | 12(92.3%) | 26(92.9%) | 197(92.9%) | 13(86.7%) | 303(92.4%) |
| Amoxicillin Trihydrate Equivalent To Amoxicillin 250mg | 5(83.3%) | 17(70.8%) | 29(96.7%) | 10(76.9%) | 24(85.7%) | 185(87.3%) | 14(93.3%) | 284)86.6% |
| Ampicillin 125mg + Cloxacillin 125mg/5ml | 6(100.0%) | 14(58.3%) | 27(90.0%) | 11(84.6%) | 21(75.0%) | 175(82.5%) | 13(86.7%) | 267(81.4%) |
| Ampicillin 250mg + Cloxacillin 250mg | 6(100.0%) | 15(62.5%) | 27(90.0%) | 9(69.2%) | 24(85.7%) | 174(82.1%) | 8(53.3%) | 263(80.2%) |
| Artemether / Lumefantrine 20/120mg | 5(83.3%) | 20(83.3%) | 29(96.7%) | 11(84.6%) | 26(92.9%) | 167(78.8%) | 14(93.3%) | 272(82.9%) |
| Artemether / Lumefantrine 15/90 Dry Suspension | 4(66.7%) | 10(41.7%) | 26(86.7%) | 10(76.9%) | 12(42.9%) | 147(69.3%) | 13(86.7%) | 222(67.7%) |
| Ascorbic Acid (Vitamin C 100mg) Tablet | 2(33.3%) | 5(20.8%) | 13(43.3%) | 5(38.5%) | 7(25.0%) | 95(44.8%) | 2(13.3%) | 129(39.3%) |
| Cetirizine Hydrochloride 10mg Tablets | 0(0.0%) | 9(37.5%) | 20(66.7%) | 1(7.7%) | 13(46.4%) | 163(76.9%) | 4(26.7%) | 210(64.0%) |
| Cetrizine Syrup 1mg/Ml | 6(100.0%) | 17(70.8%) | 25(83.3%) | 9(69.2%) | 20(71.4%) | 171(80.7%) | 14(93.3%) | 262(79.9%) |
| Chloramphenicol Palmitate 125/5ml | 5(83.3%) | 16(66.7%) | 25(83.3%) | 9(69.2%) | 9(32.1%) | 133(62.7%) | 13(86.7%) | 210(64.0%) |
| Ciprofloxacin 500mg | 4(66.7%) | 22(91.7%) | 30(100.0%) | 13(100.0%) | 26(92.9%) | 192(90.6%) | 14(93.3%) | 301(91.8%) |
| Ciprofloxacin 0.2% solution | 5(83.3%) | 10(41.7%) | 16(53.3%) | 10(76.9%) | 14(50.0%) | 86(40.6%) | 12(80.0%) | 153(46.6%) |
| Cloxacillin 125mg/5ml | 3(50.0%) | 11(45.8%) | 6(20.0%) | 8(61.5%) | 7(25.0%) | 52(24.5%) | 9(60.0%) | 96(29.3%) |
| Cloxacillin Sodium Equivalent To Cloxacillin 250mg | 4(66.7%) | 12(50.0%) | 22(73.3%) | 10(76.9%) | 21(75.0%) | 110(51.9%) | 13(86.7%) | 192(58.5%) |
| Dextrose 5% solution | 3(50.0%) | 3(12.5%) | 3(10.0%) | 4(30.8%) | 4(14.3%) | 44(20.8%) | 1(6.7%) | 62(18.9%) |
| Dextrose 50% solution | 0(0.0%) | 2(8.3%) | 7(23.3%) | 3(23.1%) | 2(7.1%) | 34(16.0%) | 9(60.0%) | 57(17.4%) |
| Diclofenac Sodium 100mg | 2(33.3%) | 6(25.0%) | 24(80.0%) | 6(46.2%) | 14(50.0%) | 189(89.2%) | 7(46.7%) | 248(75.6%) |
| Doxycycline 100mg | 4(66.7%) | 10(41.7%) | 20(66.7%) | 10(76.9%) | 21(75.0%) | 153(72.2%) | 5(33.3%) | 223(68.0%) |
| Erythromycin 125mg/5ml | 2(33.3%) | 13(54.2%) | 21(70.0%) | 10(76.9%) | 15(53.6%) | 165(77.8%) | 14(93.3%) | 240(73.2%) |
| Erythromycin 250mg tab | 6(100.0%) | 19(79.2%) | 26(86.7%) | 9(69.2%) | 22(78.6%) | 156(73.6%) | 6(40.0%) | 244(74.4%) |
| Hartmann’s Ringers Lactate solution | 2(33.3%) | 3(12.5%) | 3(10.0%) | 4(30.8%) | 4(14.3%) | 33(15.6%) | 0(0.0%) | 49(14.9%) |
| Ibuprofen 200mg Tablet | 4(66.7%) | 11(45.8%) | 18(60.0%) | 6(46.2%) | 17(60.7%) | 138(65.1%) | 11(73.3%) | 205(62.5%) |
| Ibuprofen Suspension 100mg/5ml | 4(66.7%) | 13(54.2%) | 25(83.3%) | 8(61.5%) | 18(64.3%) | 166(78.3%) | 9(60.0%) | 243(74.1%) |
| Loperamide 2mg | 3(50.0%) | 7(29.2%) | 6(20.0%) | 3(23.1%) | 7(25.0%) | 78(36.8%) | 4(26.7%) | 108(32.9%) |
| Magnesium Trisilicate 250+Dried Aluminium Hydroxide 120/Gel | 0(0.0%) | 3(12.5%) | 13(43.3%) | 9(69.2%) | 3(10.7%) | 80(37.7%) | 12(80.0%) | 120(36.6%) |
| Mannitol 20% | 3(50.0%) | 3(12.5%) | 8(26.7%) | 5(38.5%) | 4(14.3%) | 39(18.4%) | 8(53.3%) | 70(21.3%) |
| Metronidazole 0.5% | 5(83.3%) | 14(58.3%) | 15(50.0%) | 8(61.5%) | 14(50.0%) | 97(45.8%) | 11(73.3%) | 164(50.0%) |
| Metronidazole 200mg Tablets / | 4(66.7%) | 20(83.3%) | 26(86.7%) | 9(69.2%) | 27(96.4%) | 189(89.2%) | 14(93.3%) | 289(88.1%) |
| Metronidazole Suspension 100mg/5ml | 5(83.3%) | 11(45.8%) | 25(83.3%) | 10(76.9%) | 20(71.4%) | 175(82.5%) | 12(80.0%) | 268(78.7%) |
| Sodium Chloride 0.9% solution | 2(33.3%) | 6(25.0%) | 6(20.0%) | 4(30.8%) | 4(14.3%) | 35(16.5%) | 1(6.7%) | 58(17.7%) |
| Omeprazole 20mg | 4(66.7%) | 22(91.7%) | 28(93.3%) | 11(84.6%) | 28(100.0% | 201(94.8%) | 15(100.0%) | 309(94.2%) |
| ORS | 3(50.0%) | 2(8.3%) | 5(16.7%) | 3(23.1%) | 5(17.9%) | 41(19.3%) | 3(20.0%) | 62(18.9%) |
| ORS + Zinc Sulphate Monohydrate 20mg Tablets | 0(0.0%) | 0(0.0%) | 0(0.0%) | 0(0.0%) | 0(0.0%) | 1(0.5%) | 0(0.0%) | 1(0.3%) |
| Paracetamol 500mg Tablets | 4(66.7%) | 6(25.0%) | 27(90.0%) | 8(61.5%) | 8(28.6%) | 151(71.2%) | 14(93.3%) | 218(66.5%) |
| Paracetamol Suspension 120mg/5ml | 6(100.0%) | 21(87.5%) | 29(96.7%) | 13(100.0%) | 26(92.9%) | 190(89.6%) | 15(100.0%) | 300(91.5%) |
| Quinine Sulphate 300mg Tablets / Syrup 100mg/5ml | 3(50.0%) | 5(20.8%) | 14(46.7%) | 5(38.5%) | 11(39.3%) | 54(25.5%) | 5(33.3%) | 97(29.6%) |
| Quinine Sulphate Syrup 100mg/5ml | 3(50.0%) | 5(20.8%) | 11(36.7%) | 4(30.8%) | 9(32.1%) | 52(24.5%) | 7(46.7%) | 91(27.7%) |
| Sulfamethoxazole 200mg / Trimethoprim 400mg / 5ml | 5(83.3%) | 10(41.7%) | 24(80.0%) | 9(69.2%) | 16(57.1%) | 149(70.3%) | 9(60.0%) | 222(67.7%) |
| Surgical Spirit | 0(0.0%) | 0(0.0%) | 0(0.0%) | 0(0.0%) | 1(3.6%) | 5(2.4%) | 0(0.0%) | 6(1.8%) |
| Sulfamethoxazole 400mg / Trimethoprim 80mg | 3(50.0%) | 5(20.8%) | 11(36.7%) | 8(61.5%) | 13(46.4%) | 132(62.3%) | 6(40.0%) | 178(54.3%) |
| Zinc Sulfate Monohydrate BP (54.90) Equivalent To 20mg Elemental Zinc | 4(66.7%) | 10(41.7%) | 27(90.0%) | 10(76.9%) | 21(75.0%) | 149(70.3%) | 11(73.3%) | 232(70.7%) |
| Zinc Solution Supplement 10mg/5ml | 0(0.0%) | 0(0.0%) | 0(0.0%) | 0(0.0%) | 3(10.7%) | 11(5.2%) | 0(0.0%) | 14(4.3%) |

Individual selected imported medicine availability by region and overall availability in all regions

| Brands | Arua | Hoima | Jinja | Lira | Mbarara | Nakawa | Tororo | Overall |
| --- | --- | --- | --- | --- | --- | --- | --- | --- |
| Albendazole Suspension 100mg/5ml | 0(0.0%) | 7(29.2%) | 25(83.3%) | 12(92.3%) | 15(53.6%) | 173(81.6%) | 14(93.3%) | 246(75.0%) |
| Artemether / Lumefantrine 15/90 Dry Suspension | 4(66.7%) | 10(41.7%) | 26(86.7%) | 10(76.9%) | 12(42.9%) | 147(69.3%) | 13(86.7%) | 222(67.7%) |
| Cetrizine Syrup 1mg/Ml | 6(100.0%) | 17(70.8%) | 25(83.3%) | 9(69.2%) | 20(71.4%) | 171(80.7%) | 14(93.3%) | 262(79.9%) |
| Cloxacillin 125mg/5ml | 3(50.0%) | 11(45.8%) | 6(20.0%) | 8(61.5%) | 7(25.0%) | 52(24.5%) | 9(60.0%) | 96(29.3%) |
| Dextrose 5% solution | 3(50.0%) | 3(12.5%) | 3(10.0%) | 4(30.8%) | 4(14.3%) | 44(20.8%) | 1(6.7%) | 62(18.9%) |
| Hartmann’s Ringers Lactate solution | 2(33.3%) | 3(12.5%) | 3(10.0%) | 4(30.8%) | 4(14.3%) | 33(15.6%) | 0(0.0%) | 49(14.9%) |
| Sodium Chloride 0.9% solution | 2(33.3%) | 6(25.0%) | 6(20.0%) | 4(30.8%) | 4(14.3%) | 35(16.5%) | 1(6.7%) | 58(17.7%) |
| Omeprazole 20mg | 4(66.7%) | 22(91.7%) | 28(93.3%) | 11(84.6%) | 28(100.0% | 201(94.8%) | 15(100.0%) | 309(94.2%) |
| ORS | 3(50.0%) | 2(8.3%) | 5(16.7%) | 3(23.1%) | 5(17.9%) | 41(19.3%) | 3(20.0%) | 62(18.9%) |
| Paracetamol 500mg Tablets | 4(66.7%) | 6(25.0%) | 27(90.0%) | 8(61.5%) | 8(28.6%) | 151(71.2%) | 14(93.3%) | 218(66.5%) |

Table S3: Crude Median unit prices before and after the policy for local and imported products

| Brands | Local | | Imported | |
| --- | --- | --- | --- | --- |
|  | Median price (2017) | Median price (2020) | Median price (2017) | Median price (2020) |
| Albendazole 400mg Tablet | 600 | 875 | 2250 | 2850 |
| Albendazole Suspension 100mg/5ml | 90 | 90 | 325 | 350 |
| Amoxicillin Trihydrate Equivalent To Amoxicillin 125mg/5ml | 25 | 38 | 40 | 45 |
| Amoxicillin Trihydrate Equivalent To Amoxicillin 250mg | 100 | 138 | 100 | 100 |
| Ampicillin 125mg + Cloxacillin 125mg/5ml | 27 | 89 | 48 | 50 |
| Ampicillin 250mg + Cloxacillin 250mg | 163 | 200 | 200 | 200 |
| Artemether / Lumefantrine 20/120mg | 179 | 208 | 197 | 208 |
| Artemether / Lumefantrine 15/90 Dry Suspension | - | - | 175 | 208 |
| Ascorbic Acid (Vitamin C 100mg) Tablet | 50 | 113 | 50 | 100 |
| Cetirizine Hydrochloride 10mg Tablets | 50 | 100 | 200 | 200 |
| Cetrizine Syrup 1mg/Ml | 66 | 66 | 83 | 92 |
| Chloramphenicol Palmitate 125/5ml | 30 | 35 | 34 | 43 |
| Ciprofloxacin 500mg | 96 | 150 | 1350 | 1600 |
| Ciprofloxacin 0.2% solution | 1500 | 2000 | 1950 | 2375 |
| Cloxacillin 125mg/5ml | 40 | 50 | 25 | 28 |
| Cloxacillin Sodium Equivalent To Cloxacillin 250mg | 100 | 175 | 100 | 150 |
| Dextrose 5% solution | 2000 | 2500 | 2000 | 2500 |
| Dextrose 50% solution | 3350 | 3500 | 4500 | 5000 |
| Diclofenac Sodium 100mg | 40 | 65 | 1600 | 1900 |
| Doxycycline 100mg | 100 | 150 | 225 | 250 |
| Erythromycin 125mg/5ml | 35 | 40 | 35 | 43 |
| Erythromycin 250mg tab | 135 | 200 | 175 | 200 |
| Hartmann’s Ringers Lactate solution | 2000 | 2000 | 2000 | 2500 |
| Ibuprofen 200mg Tablet | 50 | 50 | 50 | 75 |
| Ibuprofen Suspension 100mg/5ml | 17 | 21 | 43 | 51 |
| Loperamide 2mg | 100 | 100 | 100 | 225 |
| Magnesium Trisilicate 250+Dried Aluminium Hydroxide 120/Gel | 35 | 39 | 43 | 49 |
| Mannitol 20% | 4000 | 3400 | 4200 | 4200 |
| Metronidazole 0.5% | 1500 | 2000 | 1750 | 2000 |
| Metronidazole 200mg Tablets / | 43 | 44 | 50 | 50 |
| Metronidazole Suspension 100mg/5ml | 29 | 33 | 35 | 43 |
| Sodium Chloride 0.9% solution | 2000 | 2500 | 2000 | 2000 |
| Omeprazole 20mg | 100 | - | 175 | 200 |
| ORS | 500 | 500 | 440 | 450 |
| ORS + Zinc Sulphate Monohydrate 20mg Tablets | 1507 | 3000 | - | - |
| Paracetamol 500mg Tablets | 38 | 38 | 133 | 150 |
| Paracetamol Suspension 120mg/5ml | 17 | 20 | 55 | 60 |
| Quinine Sulphate 300mg Tablets / Syrup 100mg/5ml | 200 | 275 | 213 | 290 |
| Quinine Sulphate Syrup 100mg/5ml | 28 | 34 | 33 | 46 |
| Sulfamethoxazole 200mg / Trimethoprim 400mg / 5ml | 19 | 32 | 41 | 48 |
| Surgical Spirit | 1550 | 1700 | 988 | 250 |
| Sulfamethoxazole 400mg / Trimethoprim 80mg | 68 | 73 | 100 | 100 |
| Zinc Sulfate Monohydrate BP (54.90) Equivalent To 20mg Elemental Zinc | 100 | 100 | 100 | 200 |
| Zinc Solution Supplement 10mg/5ml | 41 | 32 | 43 | 50 |

Table S4: Comparison of unit median prices after adjusting for inflation/deflation

| Brands | Local | | | Imported | | |
| --- | --- | --- | --- | --- | --- | --- |
|  | Median price Before | Median price (year 2020) | P-Value | Median price Before | Median price (year 2020) | P-Value |
| Albendazole 400mg Tablet | 198 | 288.75 | 0.50 | 742.5 | 940.5 | 0.251 |
| Albendazole Suspension 100mg/5ml | 29.7 | 29.7 | 1.00 | 107.3 | 115.5 | 0.50 |
| Amoxicillin Trihydrate Equivalent To Amoxicillin 125mg/5ml | 8.3 | 12.4 | 0.345 | 13.2 | 14.8 | 0.50 |
| Amoxicillin Trihydrate Equivalent To Amoxicillin 250mg | 33.0 | 45.4 | 0.50 | 33.0 | 33.0 | 1.00 |
| Ampicillin 125mg + Cloxacillin 125mg/5ml | 8.7 | 29.4 | 0.49 | 15.7 | 16.5 | 0.50 |
| Ampicillin 250mg + Cloxacillin 250mg | 53.7 | 66.0 | 0.206 | 66.0 | 66.0 | 1.00 |
| Artemether / Lumefantrine 20/120mg | 59.1 | 68.6 | 0.50 | 65.0 | 68.6 | 0.50 |
| Artemether / Lumefantrine 15/90 Dry Suspension | - | - | - | 57.6 | 68.7 | 0.009 |
| Ascorbic Acid (Vitamin C 100mg) Tablet | 16.5 | 37.2 | 0.126 | 16.5 | 33.0 | 0.897 |
| Cetirizine Hydrochloride 10mg Tablets | 16.5 | 33.0 | 0.50 | 66.0 | 66.0 | 1.00 |
| Cetrizine Syrup 1mg/Ml | 21.8 | 21.8 | 1.00 | 27.4 | 30.2 | 0.50 |
| Chloramphenicol Palmitate 125/5ml | 9.9 | 11.6 | 0.50 | 11.3 | 14.1 | 0.126 |
| Ciprofloxacin 500mg | 31.6 | 49.5 | 0.041 | 445.5 | 528.0 | 0.50 |
| Ciprofloxacin 0.2% solution | 495.0 | 660.0 | 0.50 | 643.5 | 783.8 | 0.111 |
| Cloxacillin 125mg/5ml | 13.2 | 16.5 | 0.50 | 8.3 | 9.3 | 0.382 |
| Cloxacillin Sodium Equivalent To Cloxacillin 250mg | 33.0 | 57.8 | 0.205 | 33.0 | 49.5 | 0.50 |
| Dextrose 5% solution | 660 | 825 | 0.50 | 660 | 825 | 0.50 |
| Dextrose 50% solution | 1105.5 | 1155 | 0.978 | 1485 | 1650 | 0.50 |
| Diclofenac Sodium 100mg | 13.1 | 21.6 | 0.485 | 528 | 627 | 0.374 |
| Doxycycline 100mg | 33 | 49.5 | 0.50 | 74.3 | 82.5 | 0.50 |
| Erythromycin 125mg/5ml | 11.6 | 13.3 | 0.50 | 11.6 | 14.1 | 0.197 |
| Erythromycin 250mg tab | 44.6 | 66 | 0.50 | 57.8 | 66 | 0.50 |
| Hartmann’s Ringers Lactate solution | 660 | 660 | 1.00 | 660 | 825 | 0.50 |
| Ibuprofen 200mg Tablet | 16.5 | 16.5 | 1.00 | 16.5 | 24.8 | 0.50 |
| Ibuprofen Suspension 100mg/5ml | 5.6 | 7.0 | 0.50 | 14.1 | 16.9 | 0.10 |
| Loperamide 2mg | 33 | 33 | 1.0 | 33 | 74.3 | 0.50 |
| Magnesium Trisilicate 250+Dried Aluminium Hydroxide 120/Gel | 11.6 | 12.8 | 0.205 | 14.2 | 16 | 0.374 |
| Mannitol 20% | 1320 | 1122 | 0.50 | 1386 | 1386 | 1.0 |
| Metronidazole 0.5% | 495 | 660 | 0.48 | 577.5 | 660 | 0.50 |
| Metronidazole 200mg Tablets / | 14.3 | 14.5 | 0.50 | 16.5 | 16.5 | 1.0 |
| Metronidazole Suspension 100mg/5ml | 9.4 | 10.7 | 0.75 | 11.6 | 14.1 | 0.213 |
| Sodium Chloride 0.9% solution | 660 | 825 | 0.50 | 660 | 660 | 1.0 |
| Omeprazole 20mg | 33 | - | - | 57.8 | 66 | 0.50 |
| ORS | 165 | 165 | 1.0 | 145.2 | 148.5 | 0.50 |
| ORS + Zinc Sulphate Monohydrate 20mg Tablets | 497.3 | 990 | 0.50 | - | - | - |
| Paracetamol 500mg Tablets / Suspension 120mg/5ml | 12.4 | 12.4 | 1.0 | 43.8 | 49.5 | 0.256 |
| Paracetamol Suspension 120mg/5ml | 5.5 | 6.6 | 0.237 | 18.2 | 19.8 | 0.50 |
| Quinine Sulphate 300mg Tablets | 66.0 | 90.8 | 0.205 | 70.2 | 95.7 | 0.021 |
| Quinine Sulphate Syrup 100mg/5ml | 9.3 | 11.2 | 0.386 | 10.8 | 15 | 0.322 |
| Sulfamethoxazole 200mg / Trimethoprim 400mg / 5ml | 6.2 | 10.6 | 0.287 | 13.4 | 15.9 | 0.126 |
| Surgical Spirit | 511.5 | 561 | 0.50 | 325.9 | 82.5 | 0.0003 |
| Sulfamethoxazole 400mg / Trimethoprim 80mg | 22.3 | 24.0 | 0.50 | 33 | 33 | 1.00 |
| Zinc Sulfate Monohydrate BP (54.90) Equivalent To 20mg Elemental Zinc | 33 | 33 | 1.00 | 33 | 66 | 0.025 |
| Zinc Solution Supplement 10mg/5ml | 13.5 | 10.6 | 0.622 | 14.2 | 16.5 | 0.50 |

Table S5: Comparison of median prices of lowest priced imported and local produced medicines

| Brands | Local | | | Imported | | |
| --- | --- | --- | --- | --- | --- | --- |
|  | Median price before | Median price (2020) | P-value | Median price before | Median price (2020) | P-Value |
| Albendazole 400mg Tablet | 330 | 330 | 1 | 660 | 775.5 | 0.001 |
| Albendazole Suspension 100mg/5ml | 29.7 | 29.7 | 1 | 82.5 | 99 | 0.165 |
| Amoxicillin Trihydrate Equivalent To Amoxicillin 125mg/5ml | 8.25 | 9.9 | 0.65 | 9.9 | 13.2 | 0.33 |
| Amoxicillin Trihydrate Equivalent To Amoxicillin 250mg | 33 | 33 | 1 | 33 | 33 | 1 |
| Ampicillin 125mg + Cloxacillin 125mg/5ml | 8.75 | 9.24 | 0.495 | 14.85 | 16.5 | 0.65 |
| Ampicillin 250mg + Cloxacillin 250mg | 57.75 | 66 | 0.25 | 66 | 66 | 1 |
| Artemether / Lumefantrine 20/120mg | 49.5 | 68.64 | 0.034 | 61.38 | 68.64 | 0.26 |
| Artemether / Lumefantrine 15/90 Dry Suspension | 0 | 0 | 1 | 60.39 | 71.28 | 0.189 |
| Ascorbic Acid (Vitamin C 100mg) Tablet | 16.5 | 33 | 0.026 | 16.5 | 33 | 0.165 |
| Cetirizine Hydrochloride 10mg Tablets | 16.5 | 33 | 0.046 | 66 | 66 | 1 |
| Cetrizine Syrup 1mg/Ml | 21.78 | 21.78 | 1 | 27.39 | 33 | 0.261 |
| Chloramphenicol Palmitate 125/5ml | 9.9 | 11.55 | 0.65 | 13.2 | 16.5 | 0.33 |
| Ciprofloxacin 500mg | 49.5 | 66 | 0.165 | 66 | 66 | 1 |
| Ciprofloxacin 0.2% solution | 495 | 660 | 0.016 | 660 | 825 | 0.017 |
| Cloxacillin 125mg/5ml | 13.2 | 16.5 | 0.33 | 8.25 | 9.9 | 0.65 |
| Cloxacillin Sodium Equivalent To Cloxacillin 250mg | 33 | 49.5 | 0.165 | 33 | 49.5 | 0.165 |
| Dextrose 5% solution | 660 | 825 | 0.016 | 660 | 825 | 0.017 |
| Dextrose 50% solution | 1105.5 | 1155 | 0.023 | 1320 | 1650 | 0.003 |
| Diclofenac Sodium 100mg | 16.5 | 33 | 0.165 | 231 | 264 | 0.073 |
| Doxycycline 100mg | 33 | 49.5 | 0.165 | 49.5 | 66 | 0.165 |
| Erythromycin 125mg/5ml | 9.9 | 11.55 | 0.65 | 13.2 | 16.5 | 0.33 |
| Erythromycin 250mg tab | 44.55 | 66 | 0.035 | 66 | 66 | 1 |
| Hartmann’s Ringers Lactate solution | 660 | 660 | 1 | 660 | 825 | 0.017 |
| Ibuprofen 200mg Tablet | 16.5 | 16.5 | 1 | 16.5 | 16.5 | 1 |
| Ibuprofen Suspension 100mg/5ml | 6.6 | 6.6 | 1 | 13.2 | 16.5 | 0.33 |
| Loperamide 2mg | 33 | 33 | 1 | 33 | 33 | 1 |
| Magnesium Trisilicate 250+Dried Aluminium Hydroxide 120/Gel | 8.25 | 9.9 | 0.65 | 13.53 | 16.5 | 0.397 |
| Mannitol 20% | 1320 | 1320 | 1 | 1320 | 1650 | 0.003 |
| Metronidazole 0.5% | 495 | 660 | 0.016 | 660 | 660 | 1 |
| Metronidazole 200mg Tablets / | 16.5 | 16.5 | 1 | 16.5 | 16.5 | 1 |
| Metronidazole Suspension 100mg/5ml | 9.41 | 10.73 | 0.32 | 11.55 | 14.85 | 0.33 |
| Sodium Chloride 0.9% solution | 660 | 825 | 0.017 | 660 | 660 | 1 |
| Omeprazole 20mg | 33 | 0 | 1 | 49.5 | 66 | 0.65 |
| ORS | 165 | 165 | 1 | 165 | 165 | 1 |
| ORS + Zinc Sulphate Monohydrate 20mg Tablets | 990 | 990 | 1 | 0 | 0 | 1 |
| Paracetamol 500mg Tablets | 16.5 | 16.5 | 1 | 29.7 | 33 | 0.33 |
| Paracetamol Suspension 120mg/5ml | 5.94 | 6.6 | 0.66 | 13.2 | 16.5 | 0.33 |
| Quinine Sulphate 300mg Tablets / Syrup 100mg/5ml | 66 | 82.5 | 0.165 | 66 | 92.4 | 0.064 |
| Quinine Sulphate Syrup 100mg/5ml | 9.9 | 13.2 | 0.33 | 11.55 | 13.53 | 0.798 |
| Sulfamethoxazole 200mg / Trimethoprim 400mg / 5ml | 8.25 | 10.56 | 0.37 | 13.2 | 15.18 | 0.798 |
| Surgical Spirit | 528 | 627 | 0.02 | 379.5 | 99 | 0.003 |
| Sulfamethoxazole 400mg / Trimethoprim 80mg | 33 | 33 | 1 | 33 | 33 | 1 |
| Zinc Sulfate Monohydrate BP (54.90) Equivalent To 20mg Elemental Zinc | 33 | 33 | 1 | 33 | 66 | 0.063 |
| Zinc Solution Supplement 10mg/5ml | 13.53 | 14.85 | 0.32 | 14.19 | 16.5 | 0.831 |

Table S6: International comparison of median unit prices

| Brand names | Median price/ Local 2017 | MPR | Median price/ Local 2020 | MPR | Median price/ Imported 2017 | MPR | Median price/ Imported 2020 | MPR |
| --- | --- | --- | --- | --- | --- | --- | --- | --- |
| Albendazole 400mg Tablet | 198 | 3.8 | 288.75 | 5.5 | 742.5 | 14.2 | 940.5 | 18.0 |
| Albendazole Suspension 100mg/5ml | 29.7 | 0.9 | 29.7 | 0.9 | 107.3 | 3.2 | 115.5 | 3.5 |
| Amoxicillin Trihydrate Equivalent To Amoxicillin 125mg/5ml | 8.3 | 0.5 | 12.4 | 0.7 | 13.2 | 0.8 | 14.8 | 0.9 |
| Amoxicillin Trihydrate Equivalent To Amoxicillin 250mg | 33 | 0.4 | 45.4 | 0.5 | 33 | 0.4 | 33 | 0.4 |
| Ampicillin 125mg + Cloxacillin 125mg/5ml | 8.7 | 0.5 | 29.4 | 1.6 | 15.7 | 0.9 | 16.5 | 0.9 |
| Ampicillin 250mg + Cloxacillin 250mg | 53.7 | 0.8 | 66 | 1.0 | 66 | 1.0 | 66 | 1.0 |
| Artemether / Lumefantrine 20/120mg | 59.1 | 0.2 | 68.6 | 0.2 | 65 | 0.2 | 68.6 | 0.2 |
| Artemether / Lumefantrine 15/90 Dry Suspension | - | - | - | - | 57.6 | 0.2 | 68.7 | 0.3 |
| Ascorbic Acid (Vitamin C 100mg) Tablet | 16.5 | 0.7 | 37.2 | 1.5 | 16.5 | 0.7 | 33 | 1.3 |
| Cetirizine Hydrochloride 10mg Tablets | 16.5 | 0.5 | 33 | 1.0 | 66 | 2.0 | 66 | 2.0 |
| Cetrizine Syrup 1mg/Ml | 21.8 | 1.2 | 21.8 | 1.2 | 27.4 | 1.6 | 30.2 | 1.7 |
| Chloramphenicol Palmitate 125/5ml | 9.9 | 0.3 | 11.6 | 0.4 | 11.3 | 0.4 | 14.1 | 0.5 |
| Ciprofloxacin 500mg | 31.6 | 0.2 | 49.5 | 0.4 | 445.5 | 3.3 | 528 | 3.9 |
| Ciprofloxacin 0.2% solution | 495 | 1.9 | 660 | 2.5 | 643.5 | 2.4 | 783.8 | 3.0 |
| Cloxacillin 125mg/5ml | 13.2 | 0.4 | 16.5 | 0.5 | 8.3 | 0.2 | 9.3 | 0.3 |
| Cloxacillin Sodium Equivalent To Cloxacillin 250mg | 33 | 0.4 | 57.8 | 0.8 | 33 | 0.4 | 49.5 | 0.7 |
| Dextrose 5% solution | 660 | 20.0 | 825 | 25.0 | 660 | 20.0 | 825 | 25.0 |
| Dextrose 50% solution | 1105.5 | 20.6 | 1155 | 21.6 | 1485 | 27.7 | 1650 | 30.8 |
| Diclofenac Sodium 100mg | 13.1 | 0.0 | 21.6 | 0.0 | 528 | 1.1 | 627 | 1.3 |
| Doxycycline 100mg | 33 | 0.7 | 49.5 | 1.0 | 74.3 | 1.5 | 82.5 | 1.7 |
| Erythromycin 125mg/5ml | 11.6 | 0.4 | 13.3 | 0.4 | 11.6 | 0.4 | 14.1 | 0.5 |
| Erythromycin 250mg tab | 44.6 | 0.4 | 66 | 0.6 | 57.8 | 0.5 | 66 | 0.6 |
| Hartmann’s Ringers Lactate solution | 660 | 179.8 | 660 | 179.8 | 660 | 179.8 | 825 | 224.8 |
| Ibuprofen 200mg Tablet | 16.5 | 0.7 | 16.5 | 0.7 | 16.5 | 0.7 | 24.8 | 1.0 |
| Ibuprofen Suspension 100mg/5ml | 5.6 | 0.3 | 7 | 0.4 | 14.1 | 0.8 | 16.9 | 0.9 |
| Loperamide 2mg | 33 | 1.0 | 33 | 1.0 | 33 | 1.0 | 74.3 | 2.2 |
| Magnesium Trisilicate 250+Dried Aluminium Hydroxide 120/Gel | 11.6 | 0.6 | 12.8 | 0.7 | 14.2 | 0.8 | 16 | 0.9 |
| Mannitol 20% | 1320 | 35.6 | 1122 | 30.3 | 1386 | 37.4 | 1386 | 37.4 |
| Metronidazole 0.5% | 495 | 27.0 | 660 | 36.0 | 577.5 | 31.5 | 660 | 36.0 |
| Metronidazole 200mg Tablets / | 14.3 | 0.6 | 14.5 | 0.6 | 16.5 | 0.7 | 16.5 | 0.7 |
| Metronidazole Suspension 100mg/5ml | 9.4 | 0.4 | 10.7 | 0.5 | 11.6 | 0.5 | 14.1 | 0.7 |
| Sodium Chloride 0.9% solution | 660 | 179.8 | 825 | 224.8 | 660 | 179.8 | 660 | 179.8 |
| Omeprazole 20mg | 33 | 0.7 | - |  | 57.8 | 1.2 | 66 | 1.4 |
| ORS | 165 | 0.8 | 165 | 0.8 | 145.2 | 0.7 | 148.5 | 0.7 |
| ORS + Zinc Sulphate Monohydrate 20mg Tablets | 497.3 | 0.5 | 990 | 0.9 | - |  | - |  |
| Paracetamol 500mg Tablets | 12.4 | 0.8 | 12.4 | 0.8 | 43.8 | 2.7 | 49.5 | 3.1 |
| Paracetamol Suspension 120mg/5ml | 5.5 | 0.3 | 6.6 | 0.3 | 18.2 | 1.0 | 19.8 | 1.0 |
| Quinine Sulphate 300mg Tablets | 66 | 0.3 | 90.8 | 0.4 | 70.2 | 0.3 | 95.7 | 0.4 |
| Quinine Sulphate Syrup 100mg/5ml | 9.3 | 0.3 | 11.2 | 0.4 | 10.8 | 0.4 | 15 | 0.5 |
| Sulfamethoxazole 200mg / Trimethoprim 400mg / 5ml | 6.2 | 0.4 | 10.6 | 0.7 | 13.4 | 0.9 | 15.9 | 1.0 |
| Surgical Spirit | 511.5 | 25.8 | 561 | 28.3 | 325.9 | 16.4 | 82.5 | 4.2 |
| Sulfamethoxazole 400mg / Trimethoprim 80mg | 22.3 | 0.5 | 24 | 0.5 | 33 | 0.7 | 33 | 0.7 |
| Zinc Sulfate Monohydrate BP (54.90) Equivalent To 20mg Elemental Zinc | 33 | 0.8 | 33 | 0.8 | 33 | 0.8 | 66 | 1.6 |
| Zinc Solution Supplement 10mg/5ml | 13.5 | 0.9 | 10.6 | 0.7 | 14.2 | 0.9 | 16.5 | 1.0 |

Table S7: Mean availability of local manufactured medicines

| Characteristics | | Wholesale Pharmacy (%) | Retail Pharmacy (%) | Both wholesale and retails (%) | Overall | P value |
| --- | --- | --- | --- | --- | --- | --- |
| Region | Central-Nakawa | 37.0 | 30.4 | 36.4 | 31.9 | <0.0001 |
|  | Southwestern-Mbarara | 39.4 | 31.5 | 0.0 | 35.5 |
|  | West Nile-Arua | 26.7 | 36.4 | 0.0 | 29.9 |
|  | Western-Hoima | 47.3 | 37.4 | 56.8 | 43.2 |
|  | Northern-Lira | 35.2 | 37.7 | 0.0 | 36.5 |
|  | Eastern-Tororo | 45.5 | 44.6 | 47.7 | 45.2 |
|  | South eastern-Jinja | 41.1 | 34.8 | 43.9 | 37.8 |
|  |  |  |  |  |  |  |
| VEN classification | Vital | 38.1 | 31.4 | 45.3 | 33.7 | <0.0001 |
|  | Essential | 33.7 | 25.0 | 40.5 | 27.9 |
|  | Necessary | 58.8 | 54.2 | 60.0 | 55.7 |
|  |  |  |  |  |  |  |
| Formulation | Tablets/capsules | 38.6 | 34.2 | 46.8 | 35.8 | 0.0011 |
|  | Oral suspensions/syrups | 27.1 | 18.7 | 34.4 | 21.5 |
|  | Parenteral preparations | 62.4 | 51.0 | 62.5 | 54.6 |

Table S8: Mean Availability of imported medicines

| Characteristics |  | Wholesale Pharmacy (%) | Retail Pharmacy (%) | Both wholesale and retails (%) | Overall | P value |
| --- | --- | --- | --- | --- | --- | --- |
| Region | Central-Nakawa | 51.2 | 58.6 | 50.0 | 57.0 | <0.0001 |
|  | Southwestern-Mbarara | 50.0 | 51.6 | 0 | 50.8 |
|  | West Nile-Arua | 54.0 | 60.2 | 0 | 56.1 |
|  | Western-Hoima | 40.2 | 47.5 | 18.2 | 42.6 |
|  | Northern-Lira | 57.6 | 57.8 | 0 | 57.7 |
|  | Eastern-Tororo | 56.8 | 61.9 | 52.3 | 59.2 |
|  | South eastern-Jinja | 59.5 | 61.4 | 57.6 | 60.4 |
| VEN classification | Vital | 53.9 | 59.1 | 52.0 | 57.4 | <0.0001 |
|  | Essential | 52.2 | 59.9 | 48.8 | 57.4 |
|  | Necessary | 36.4 | 47.1 | 33.3 | 43.6 |
|  |  |  |  |  |  |  |
| Formulation | Tablets/capsules | 56.6 | 67.4 | 54.0 | 63.9 | 0.0012 |
|  | Oral suspensions/syrups | 58.9 | 63.2 | 53.3 | 61.7 |
|  | Parenteral preparations | 23.6 | 23.5 | 27.1 | 23.6 |

Table S9: Impact of 12% increase in import verification fees on price of medicines

| **Impact on price** | **Frequency n=7** |
| --- | --- |
| There was definitely no difference in prices before and after BUBU though the prices are very competitive in the market. | 2 |
| Prices of the items are still the same or even higher in some instances | 2 |
| The local manufacturers are putting prices like those of imported products and yet we thought we would get a much lower price | 1 |
| The policy led to increase in prices to patients of the 37 products and others | 1 |
| The general public has not enjoyed any reduction in the cost as a result of that policy | 1 |

**Table S10: Impact of 12% increase in import verification fees on availability of drugs**

| **Impact on availability** | **Frequency n=7** |
| --- | --- |
| The local manufacturers import raw materials they use in manufacturing which affects availability of drugs and production | 3 |
| Local manufacturers cannot deliver what you want and you have to wait for some weeks or months | 2 |
| The effort has been put in making sure that the 37 products are available by the local manufacturers | 1 |
| As far as availability is concerned, in some cases these drugs are not available in the quantity required by the country | 1 |
| The 12% increase was too high and some products the local producers were producing were dropped from importation | 1 |
| The cost of local products is high and it is the end user who is suffering paying high costs affecting availability | 1 |

**S11: Key informant interview guide**

1. Institution of the key informant
2. Pharmaceutical industry
3. Uganda Pharmaceutical manufacturers association
4. Uganda Pharmacy Owners Association
5. Manager National Medical stores and Joint medical stores
6. Importer (those importing more than 5 of the selected 37 essential medicines)
7. What is your current position and how long have you been in your current position?

(Allow me draw your attention to 12 percent increase in import verification fees that was imposed by government of Uganda in 2016 to date)

1. What were the reasons for increasing the import verification fees to 12%? (*What were the intended objectives?)*
2. To what extent do you think the objectives of the increase have been met? Hint if not mentioned, draw the attention of the key informant on impact of the increase on capacity and volume of local production, cost and availability of the medicines.
3. What are some of the challenges if any that hinders the achievement of the objectives of increase in the import verification fees?
4. What recommendations would you make regarding the verification fees?
5. What other comment do you have regarding the import verification fees

Thank you very much
